# Prediction performance of scoring systems after out-of-hospital cardiac arrest: A systematic review and meta-analysis

**DOI:** 10.1371/journal.pone.0293704

**Published:** 2024-02-01

**Authors:** Boldizsár Kiss, Rita Nagy, Tamás Kói, Andrea Harnos, István Ferenc Édes, Pál Ábrahám, Henriette Mészáros, Péter Hegyi, Endre Zima

**Affiliations:** 1 Heart and Vascular Centre, Semmelweis University, Budapest, Hungary; 2 Centre for Translational Medicine, Semmelweis University, Budapest, Hungary; 3 Heim Pál National Pediatric Insitute, Budapest, Hungary; 4 Institute for Translational Medicine, Medical School, University of Pécs, Pécs, Hungary; 5 Mathematical Institute, Budapest University of Technology and Economics, Budapest, Hungary; 6 Department of Biostatistics, University of Veterinary Medicine, Budapest, Hungary; 7 Institute for Pancreatic Diseases, Semmelweis University, Budapest, Hungary; Stony Brook University Renaissance School of Medicine, UNITED STATES

## Abstract

**Introduction:**

Ongoing changes in post resuscitation medicine and society create a range of ethical challenges for clinicians. Withdrawal of life-sustaining treatment is a very sensitive, complex decision to be made by the treatment team and the relatives together. According to the guidelines, prognostication after cardiopulmonary resuscitation should be based on a combination of clinical examination, biomarkers, imaging, and electrophysiological testing. Several prognostic scores exist to predict neurological and mortality outcome in post-cardiac arrest patients. We aimed to perform a meta-analysis and systematic review of current scoring systems used after out-of-hospital cardiac arrest (OHCA).

**Materials and methods:**

Our systematic search was conducted in four databases: Medline, Embase, Central and Scopus on 24th April 2023. The patient population consisted of successfully resuscitated adult patients after OHCA. We included all prognostic scoring systems in our analysis suitable to estimate neurologic function as the primary outcome and mortality as the secondary outcome. For each score and outcome, we collected the AUC (area under curve) values and their CIs (confidence iterval) and performed a random-effects meta-analysis to obtain pooled AUC estimates with 95% CI. To visualize the trade-off between sensitivity and specificity achieved using different thresholds, we created the Summary Receiver Operating Characteristic (SROC) curves.

**Results:**

24,479 records were identified, 51 of which met the selection criteria and were included in the qualitative analysis. Of these, 24 studies were included in the quantitative synthesis. The performance of CAHP (Cardiac Arrest Hospital Prognosis) (0.876 [0.853–0.898]) and OHCA (0.840 [0.824–0.856]) was good to predict neurological outcome at hospital discharge, and TTM (Targeted Temperature Management) (0.880 [0.844–0.916]), CAHP (0.843 [0.771–0.915]) and OHCA (0.811 [0.759–0.863]) scores predicted good the 6-month neurological outcome. We were able to confirm the superiority of the CAHP score especially in the high specificity range based on our sensitivity and specificity analysis.

**Conclusion:**

Based on our results CAHP is the most accurate scoring system for predicting the neurological outcome at hospital discharge and is a bit less accurate than TTM score for the 6-month outcome. We recommend the use of the CAHP scoring system in everyday clinical practice not only because of its accuracy and the best performance concerning specificity but also because of the rapid and easy availability of the necessary clinical data for the calculation.

## Introduction

Sudden cardiac arrest (SCA) is one of the most common causes of cardiovascular death [1]. Survival depends on prehospital and in-hospital factors. All out-of-hospital cardiac arrest (OHCA) patients, even those who have reached the return of spontaneous circulation (ROSC) are admitted to the hospital and need intensive care for a certain period of time. Critical state patients who need complex intensive and multiorgan supportive care after cardiac arrest have the worst outcome. Even if clinical survival is achieved, it is not necessarily accompanied by good neurological outcome [2].

The duration of post resuscitation care in the intensive care unit (ICU) ranges from hours to weeks, depending on the support demand and the target organ function. The structural and functional state of the central nervous system is the main determinant of survival. Long intensive care means the highest cost (€120,000–168,000) and resource overuse (human and material) in the healthcare system and the greatest psychological trauma for the relatives [3,4].

Ongoing changes in medicine and society create a range of challenges for the healthcare system. Besides, it is a hard time for the relatives of the patient, who need objective information about the odds. Assessing which patients survive with good neurological function is a major challenge for the clinician during the treatment of such patients.

Sixty-six percent of patients admitted to the ICU following OHCA die of a neurological injury in hospital. However, most of the definitive deaths after successful resuscitation are due to active withdrawal of life-sustaining treatment (WLST) in cases where the medical team identifies a poor neurological outcome. Accurate prognostication is fundamental to avoid inappropriate WLST and the application of costly intensive resources in cases of futility [5,6]. WLST is a very sensitive, complex decision to be made by the treatment team and the relatives together. It is sensitive for the relatives due to emotional and religious reasons as well. The introduction of therapeutic targeted temperature management and the time spent on sedatives and neuromuscular blockades make these decisions even more difficult and prolong the decision time. According to the guidelines, prognostication after cardiopulmonary resuscitation should be based on a combination of clinical examination, biomarkers, imaging and electrophysiological testing [3,7].

There are several prognostic scoring systems (PSS) to predict the prognosis (neurological and mortality outcome) of OHCA patients. These scores (predictive factors and accuracy) and the reported resuscitated patient population are highly heterogeneous. To date, few prediction scoring systems have been useful, feasible and reliable for accurately estimating the neurologic outcome in the early phase of intensive care after admission. If we seriously consider using prediction scoring systems in clinical practice, we must look for a system that can predict poor outcome with a specificity of around 100% [8]. Otherwise, we can lose”late awakening” patients by using incorrectly chosen decision support systems [9].

Currently, there is no consensus on which scoring system can be used safely as a decision support system in daily clinical practice. We aimed to perform a meta-analysis and systematic review of current scoring systems used after OHCA to identify the best performing score system.

## Materials and methods

We report our systematic review and meta-analysis based on the PRISMA (Preferred Reporting Items for Systematic Reviews and Meta-Analyses) 2020 guideline (S1 Table) [10], following the recommendation of the Cochrane Handbook [11]. The study protocol was registered on PROSPERO [CRD42021284545] [12].

### Eligibility criteria

The research question was formulated by using the Population, Intervention, Comparator, and Outcomes (PICO) framework [13]. Cohort studies and clinical trials were eligible; however, case reports, case series reports, conference abstracts, and articles with no original data were excluded from our systematic review.

The patient population consisted of successfully resuscitated adult patients after OHCA. We accepted prognostic scoring systems or prediction models suitable to estimate the clinical outcome from easily available parameters after admission to the ICU. We excluded all the prognostic scores or prediction models based on mixed (OHCA and IHCA) population.

The primary outcome was the neurologic function after resuscitation, which was categorized with the Cerebral Performance Category (CPC) as good (CPC 1–2) or poor (CPC 3–5) according to standard intensive care unit practice [2,14]. As a secondary outcome, we investigated mortality at different time points after cardiac arrest.

### Information sources, search strategy and selection process

We conducted the systematic search in four databases: MEDLINE (via PubMed), Embase, Cochrane Central Register of Controlled Trials (CENTRAL) and Scopus on April 24^th^ 2023. The complete search strategy is reported in S1 File. Two independent review authors (BK and HM) performed the selection process with reference management software (Endnote X9.3.3, Clarivate Analytics, 2020). Duplicates were removed automatically and manually. Disagreements were resolved by a third reviewer (RN).

Records were selected for meta-analysis if OHCA patients were enrolled consecutively; if prediction scores were used to predict neurological outcome or mortality; and if sensitivity and specificity values, the absolute number of true positive (TP), false negative (FN), false positive (FP) and true negative (TN), and/or area under the curve (AUC) were reported with confidence interval. Only full-text articles were included in our systematic review (qualitative synthesis) and meta-analysis (quantitative synthesis).

### Data collection process

Data from eligible articles were collected independently by two authors (BK and HM) on a standardized data collection sheet (Microsoft Excel for Mac, Microsoft, 2022). The accuracy of the data was validated by a third reviewer (RN).

### Data items

The following data were extracted from each eligible article: title; first author; the year of publication; Digital Object Identifier (DOI); study site; study period; study design; recruitment period; gender; age and initial rhythm ratios in the populations; application of targeted temperature management (TTM) therapy; serum lactate and creatinine on admission; pH (potential of hydrogen) on admission; time factors of the cardiopulmonary resuscitation (no-flow and low-flow time), parameters used by different prediction models or scoring systems.

In addition to the sensitivity and specificity values for various thresholds, the absolute numbers of TP, FN, FP and TN, AUC with confidence interval (CI), cut-off value, and clinical end-points were collected.

### Assessment of the risk of bias in the study

The Prediction model Risk Of Bias ASsessment Tool (PROBAST) was used to assess the risk of bias and the applicability of primary studies following the recommendation of the Cochrane Collaboration [15]. Two authors (BK and HM) performed the risk of bias (ROB) assessment independently. Any disagreement was resolved by consensus.

### Synthesis methods

Statistical analyses were carried out using the R statistical software (version 4.1.2.) and the R script of the online tool described by Freeman [16]. For all statistical analyses, a p-value of less than 0.05 was considered significant.

After collection of the AUC values and their CIs for each score and outcome, when there were at least three related cohorts then the meta-analysis was performed. We estimated the standard deviations of the AUC values from the CIs. When a confidence interval was not available, we used the formula introduced by Hanley et al. [17]. Several studies analysed two or three prognostic scores. To account for these correlations, we fitted a multivariable model using the rma.mv() function of the metafor R package. To resolve the problem caused by the unknown correlations, we applied the robust correction of Pustejovsky implemented in the coef_test() function of the clubSandwhich R package [18]. As the result, the concomitant AUC values and CIs were dispatched. Moreover, we repeated the approach under several between-study and within-study correlation assumptions. All of the sensitivity runnings provided essentially the same pooled AUC values and comparison p-values. In the case of all the other AUC meta-analyses, we applied the classical univariate inverse-variance random-effects meta-analysis with the restricted maximum likelihood. Heterogeneity was assessed by calculating the univariate I^2^ measure and its confidence interval and performing the Cochrane Q test. Even when the pooled estimate was created using the multivariate approach, we calculated the I^2^ values provided by the univariate method. I^2^ values of 25%, 50%, and 75% were considered low, moderate, and high heterogeneity, respectively (11). The following categories were used to interpret discriminatory performance of AUC: ≥0.9 = excellent; 0.8–0.9 = good; 0.7–0.8 = fair; 0.6–0.7 = poor and 0.5–0.6 = fail [19,20].

To get a better insight into the diagnostic performance of CAHP and OHCA scores, we collected the total number of patients with “CPC 1–2” and “CPC 3–5” status and sensitivity and specificity values along with the corresponding thresholds. From these data, we calculated two-by-two contingency tables for each threshold containing the true positive, false positive, false negative, and true negative values. To consider the dependency between sensitivity and specificity, we created Summary Receiver Operating Characteristic (SROC) curves along with CIs using the method introduced by Steinhauser and Rücker. The advantage of this relatively new approach is that it handles the correlation between contingency tables from the same studies corresponding to different thresholds. However, the underlying model has a large number of parameters, and the threshold values need to be known. [21]. For this reason, we also fitted the SROC curve using the non-Bayesian version of the approach introduced by Rutter and Gatsonis [22]. We randomly chose a threshold from each study using a random selection that ensures that the chosen thresholds are substantially different from each other. We fitted the model to the corresponding (random) dataset. We repeated this procedure 16 times.

The paper of Harbord shows that the method of Rutter and Gatsonis [23] is mathematically equivalent to the bivariate model of Reitsma and Chu [24,25] focusing on the pooled sensitivity and specificity. The pooled sensitivity and specificity are meaningful only if all the input data correspond to the same threshold. For thresholds 200 and 150 in case of the CAHP, and 60 and 40 in case of the OHCA, we calculated pooled sensitivity and specificity, and we visualized it on ROC plot.

When raw data was available, we calculated certain outcomes that were not published in the original studies, e.g., we calculated sensitivity and specificity values corresponding to numerous different thresholds.

## Results

### Search and selection

**Fig 1:**
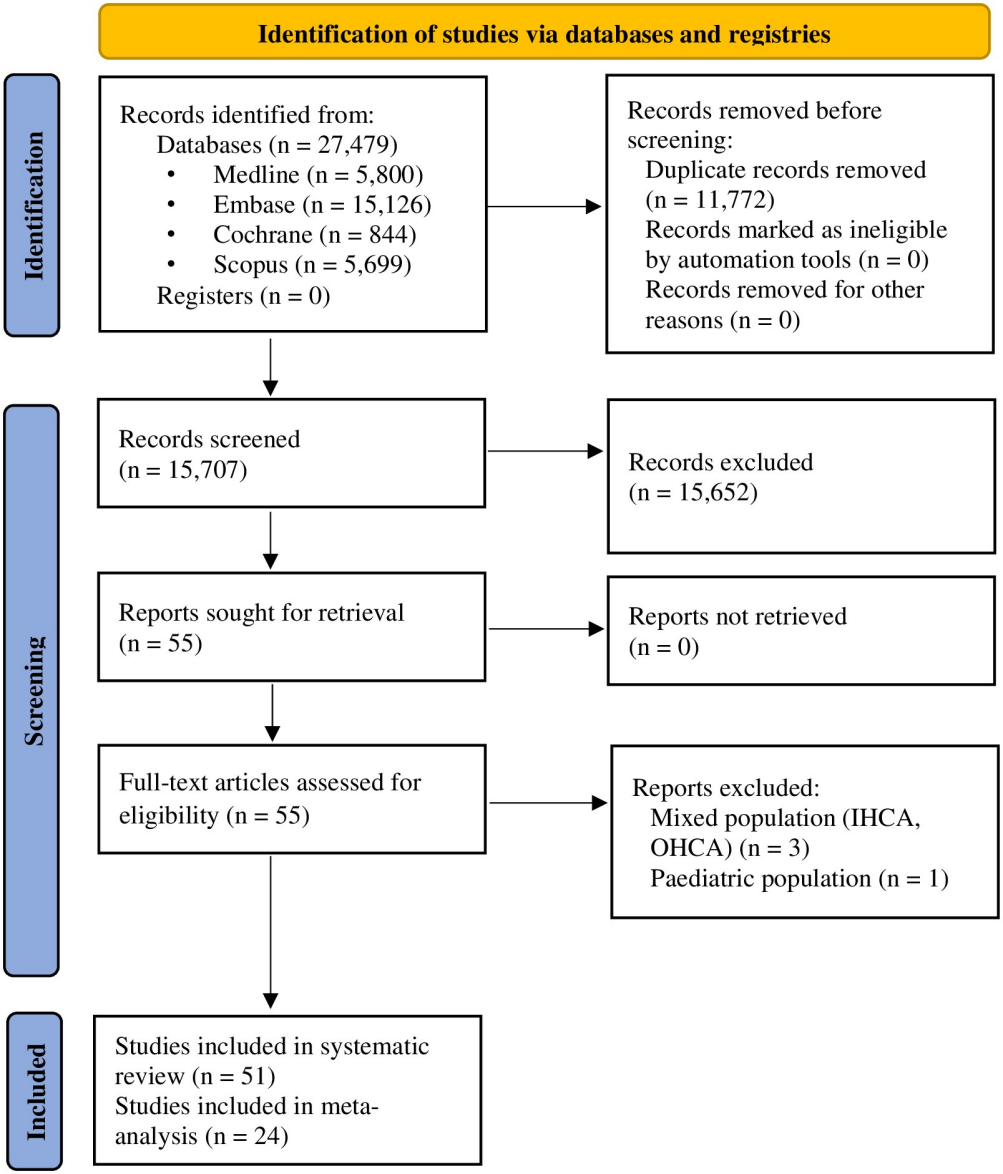
PRISMA 2020 flowchart representing the study selection process [10]. *IHCA*: *in-hospital-cardiac arrest*; *OHCA*: *out-of-hospital cardiac arrest*; *PRISMA*: *Preferred Reporting Items for Systematic Reviews and Meta-Analyses*.

The systematic search identified a total of 27,479 records in four databases. After automatic and manual duplicate removal, 15,707 records were screened, and finally, 51 full-text papers were included in the qualitative synthesis, and 24 papers were included in the quantitative analysis. The selection process is shown in Fig 1.

### Systematic review

In the 51 identified articles, data of 86,321 patients data were used to develop and validate 36 scoring systems or prediction models [26–76]. These scoring systems were developed and validated mainly in Asian or Caucasian populations. There was no prognostic scoring system for Latin American and African populations. All studies included female and male participants in widely varying proportions (male: 56–88%). The cohorts varied considerably at the extent to which they used therapeutic hypothermia or targeted temperature management as a part of post-resuscitation intensive care (from 4 to 100%). In the case of 33 out of 51 articles, data collection began before 2013 (S2 Table).

As a part of the systematic review, we summarized all the variables used by different prediction scoring systems. The number of variables in different scoring systems ranged from 1 to 18, with a median of six variables per scoring system. The initial rhythm was the most common variable (in 25 PSS), followed by age (in 18 PSS), no-flow time (in 14 PSS), low-flow time (in 13 PSS), pH (in 13 PSS), and the witness at the time of arrest (in 12 PSS). Of all the identified scoring systems, 13 were externally validated in the same study (S3 Table).

### Basic characteristics of studies included in the meta-analysis

The basic characteristics of the 24 included articles included in the meta-analysis are detailed in Tables 1 and 2 [26,27,29,32,33,36,38,41,42,46–48,54–57,63,65,66,68,69,72,73,76]. The included cohorts contain data of 13,261 OHCA patients. We were able to include the following 7 score systems in the meta-analysis: CAHP (**C**ardiac **A**rrest **H**ospital **P**rognosis), C-GRApH (**C**: coronary artery disease, known pre‐arrest; **G**: glucose; **R**: rhythm of arrest not ventricular tachycardia or fibrillation; **A**: age; **pH**: arterial pH), NULL-PLEASE (**N**onshockable rhythm, **U**nwitnessed arrest, **L**ong no-flow or **L**ong low-flow period, blood **p**H, **L**actate, **E**nd-stage chronic kidney disease on dialysis, **A**ge, **S**till resuscitation, and **E**xtracardiac cause), OHCA (**O**ut-of-**H**ospital **C**ardiac **A**rrest), rCAST (**r**evised post-**C**ardiac **A**rrest **S**yndrome for **T**herapeutic hypothermia), SOFA (**S**equential **O**rgan **F**ailure **A**ssessment) and TTM (**T**argeted **T**emperature **M**anagement). The most common clinical endpoint was neurological outcome at hospital discharge.

**Table 1:** Basic characteristics of studies included in the meta-analysis.

First author, year	Study site	Study type	Study design	Study period	Investigated score systems	Number of patients (N)	Male (%)	Age (year)
Adrie et al., [26] 2006 (dev)	France	multicenter	prospective	1999–2003	OHCA	130	72	55 (47–69)^b^
Adrie et al., [26] 2006 (val)	France	multicenter	prospective	2003–2005	OHCA	210	80	56 (45–69)^b^
Bae et al., [27] 2021	Republic of Korea	single-center	retrospective	2014–2016	CAHP, C-GRApH, OHCA	671	68	63 (52–74)^b^
Blatter et al., [29] 2023	Switzerland	single-center	prospective	2012–2022	CAHP, OHCA,	687	72	66 (56–76)^b^
Chen et al., [32] 2022	Taiwan	single-center	retrospective	2015–2021	rCAST	108	61	66 (56–78)^b^
Choi et al., [33] 2018	Republic of Korea	single-center	retrospective	2010–2013	OHCA, SOFA	173	69	53 (±15) ^a^
Gue et al., [36] 2020 (dev)	United Kingdom	multicenter	retrospective	2015–2018	NULL-PLEASE	300	71	N/A
Gue et al., [36] 2020 (val)	United Kingdom	multicenter	prospective	2015–2018	NULL-PLEASE	400	75	N/A
Heo et al., [38] 2022	Republic of Korea	multicenter	retrospective	2015–2018	CAHP, C-GRApH, NULL-PLEASE, OHCA, rCAST, TTM	1186	71	58 (47–70)^b^
Hunziker et al., [41] 2011	USA	multicenter	retrospective	2006–2008	OHCA	128	69	62 (52–77)^b^
Isenschmid et al., [42] 2019	Switzerland	single-center	prospective	2012–2017	CAHP, OHCA	349	73	65 (56–75)^b^
Kägi et al., [46] 2020	Switzerland	single-center	retrospective	2016	TTM	100	N/A	N/A
Kiehl et al., [47] 2017 (dev)	USA	multicenter	prospective	2008–2012	C-GRApH	122	68	60 (±16) ^a^
Kiehl et al., [47] 2017 (val)	USA	multicenter	prospective	2012–2014	C-GRApH	344	56	62 (±15) ^a^
Kim et al., [48] 2020	Republic of Korea	single-center	retrospective	2009–2017	CAHP, C-GRApH, OHCA	311	71	55 (44–69)^b^
Leusher et al., [54] 2019	Switzerland	single-center	prospective	2012–2017	CAHP, OHCA	336	72	64 (±14) ^a^
Martinell et al., [55] 2017	Australia, Europe ^c^	RCT	multicentre	2010–2013	CAHP, OHCA, TTM	933	81	65 (57–73)^b^
Matsuda et al., [56] 2020	Japan	single-center	retrospective	2015–2018	SOFA	231	76	61 (±17)^a^
Maupain et al., [57] 2016 (dev)	France	multicenter	prospective	2011–2012	CAHP	819	69	62 (±15)^a^
Maupain et al., [57] 2016 (val.1)	France	multicenter	retrospective	2007–2010	CAHP	367	71	62 (±15)^a^
Maupain et al., [57] 2016 (val.2)	France	multicenter	prospective	2013–2014	CAHP	1,129	67	62 (±16)^a^
Pareek et al. [63] 2020 (dev)	UK	multicenter	retrospective	2012–2017	CAHP, OHCA, TTM	373	74	64 (52–75)^b^

parameters expressed as mean with standard deviation (^a^), or median with interquartile range (^b^).

^c^ Denmark, Italy, Luxembourg, Netherlands, Norway, Sweden, Switzerland, United Kingdom.

dev: Development cohort, N/A: Not available, RCT: Randomized controlled trial, UK: United Kingdom, USA: United States of America, val: Validation cohort.

**Table 2:** Basic characteristics of post-cardiac-arrest patients in studies included in the meta-analysis.

First author, year	Shockable initial rhythm (%)	No-flow time (min)	Low-flow time (min)	Serum lactate (mmol/L) ^c^	pH ^c^	Serum creatinine (μmol/L) ^c^	TTM (%)
Adrie et al., [26] 2006 (dev)	42	6 (3–10)**	15 (10–25)**	6.7 (3.7–11.0)**	7.25 (7.15–7.34)**	118 (99–147)**	11
Adrie et al., [26] 2006 (val)	38	7 (3–10)**	20 (12–33)**	6.2 (3.2–11.3)**	N/A	120 (100–152)**	74
Bae et al., [27] 2021	30	2 (1–5)**	20 (10–32)**	8.7 (5.5–13.1)**	7.16 (6.99–7.32)**	106 (88–150)**	52
Blatter et al., [29] 2023	53	0 (0–5)**	15 (10–25)**	5.6 (2.9–9.0)**	7.25 (7.12–7.33)**	99 (78–124)**	51
Chen et al., [32] 2022	28	N/A	28 (15–41)**	6.2 (4.3–9.6)**	7.36 (7.26–7.43)**	N/A	100
Choi et al., [33] 2018	34	6 (2–11)**	22 (16–33)**	7.9 (5.8–10.5)**	7.29 (7.21–7.36)**	88 (71–106)**	100
Gue et al., [36] 2020 (dev)	89	N/A	N/A	N/A	N/A	N/A	N/A
Gue et al., [36] 2020 (val)	63	N/A	N/A	N/A	N/A	N/A	N/A
Heo et al., [38] 2022	36	1 (0–6)**	25 (14–38)**	9.1 (4.3–12.6)**	7.11 (6.95–725)**	113 (93–137)**	100
Hunziker et al., [41] 2011	23	4 (1–6)**	20 (10–27)**	9.7 (4.3–14.6)**	N/A	150 (106–256)**	34
Isenschmid et al., [42] 2019	55	0 (0–8)**	15 (10–15)**	6.6 (4.6–9.7)**	7.26 (7.19–7.33)**	N/A	57
Kägi et al., [46] 2020	34	N/A	N/A	N/A	N/A	N/A	100
Kiehl et al., [47] 2017 (dev)	67	N/A	30 (±17)*	N/A	7.17 (± 0.15)*	N/A	100
Kiehl et al., [47] 2017 (val)	38	N/A	27 (± 7)*	N/A	7.15 (± 0.19)*	N/A	100
Kim et al., [48] 2020	37	5 (0–10)**	25 (14–35)**	10.0 (5.0–14.2)**	7.13 (6.91–7.28)**	107 (88–135)**	100
Leusher et al., [54] 2019	56	4 (±6)*	19 (±15)*	N/A	N/A	N/A	100
Martinell et al., [55] 2017	78	N/A	N/A	N/A	N/A	N/A	N/A
Matsuda et al., [56] 2020	68	N/A	N/A	11.0 (± 5.15)*	7.07 (6.89–7.26)**	N/A	62
Maupain et al., [57] 2016 (dev)	44	5 (±6)*	23 (±15)*	N/A	7.19 (±0.18)*	136 (±100)*	N/A
Maupain et al., [57] 2016 (val.1)	54	5 (±6)*	19 (±14)*	N/A	7.21 (±0.16)*	135 (±126)*	N/A
Maupain et al., [57] 2016 (val.2)	54	5 (±6)*	22 (±14)*	N/A	7.18 (±0.18)*	138 (±105)*	N/A
Pareek et al. [63] 2020 (dev)	70	2 (0–7)**	25 (17–38)**	4.9 (2.4–8.9)**	7.21 (7.08–7.30)**	108 (86–134)**	N/A
Pareek et al. [63] 2020 (val.1)	82	N/A	N/A	3.60 (1.9–7.4)**	7.27 (7.17–7.34)**	104 (85–125)**	N/A
Pareek et al. [63] 2020 (val.2)	78	N/A	N/A	4.93 (2.8–8.4)**	7.24 (7.07–7.35)**	95 (73–123)**	N/A
Pham et al., [65] 2021	82	4 (±5)*	19 (±13)*	4.8 (±4.4)*	7.24 (±0.13)*	105 (±51)*	90
Sauneuf et al., [66] 2020	39	5 (2–10)**	20 (13–27)**	4.5 (2.5–8.5)**	7.30 (7.20–7.39)**	121 (95–147)**	45
Shibahashi et al., [68] 2020	22	10 (4–14)**	30 (16–41)**	N/A	6.97 (6.84–7.13)**	98 (80–135)**	N/A
Tuchida et. al. [69] 2021	28	N/A	N/A	N/A	N/A	N/A	53
Vedamurthy et al., [72] 2021	N/A	N/A	N/A	7.7 (±4.1)*	N/A	202 (±185)*	N/A
Wu et al., [73] 2022	8	N/A	N/A	N/A	N/A	N/A	N/A
Yoon et al., [76] 2018	25	N/A	N/A	N/A	N/A	N/A	100

parameter estimates are expressed as mean with standard deviation (^a^), or median with interquartile range (^b^).

^c^ initial values after hospital admission.

dev: Development cohort, N/A: Not available, pH: Potential of hydrogen, TTM: Targeted temperature management, val: Validation cohort.

Studies were conducted in 15 countries between 1999 and 2022. The cohorts were quite different and had a wide range of the basic characteristics: age (53–81); gender (male: 56–82%); initial rhythm (shockable initial rhythm: 8–89%); no-flow (0–10 minutes) and low-flow (15–30 minutes) times; serum lactate (3.6–11.0 mmol/L), pH (6.97–7.36) and creatinine (88–202 μmol/L) values after ICU admission; and application of targeted temperature management (11–100%).

### Risk of bias assessment

We assessed study quality using the PROBAST checklist. A detailed assessment for each domain and the graphical presentation of ROB and applicability are presented in S1 Fig and S4 Table. Overall, ROB was “low” in 33 studies, “high” in 16 studies, and “unclear” in 2 studies. Applicability was “low concern” in 42 studies, “high concern” in 8 studies, and “unclear” in 1 study. Within the risk of bias assessment and the applicability domains, “low” risk of bias was observed in most domains.

### Performance of scores to predict neurological outcome at hospital discharge

**Fig 2:**
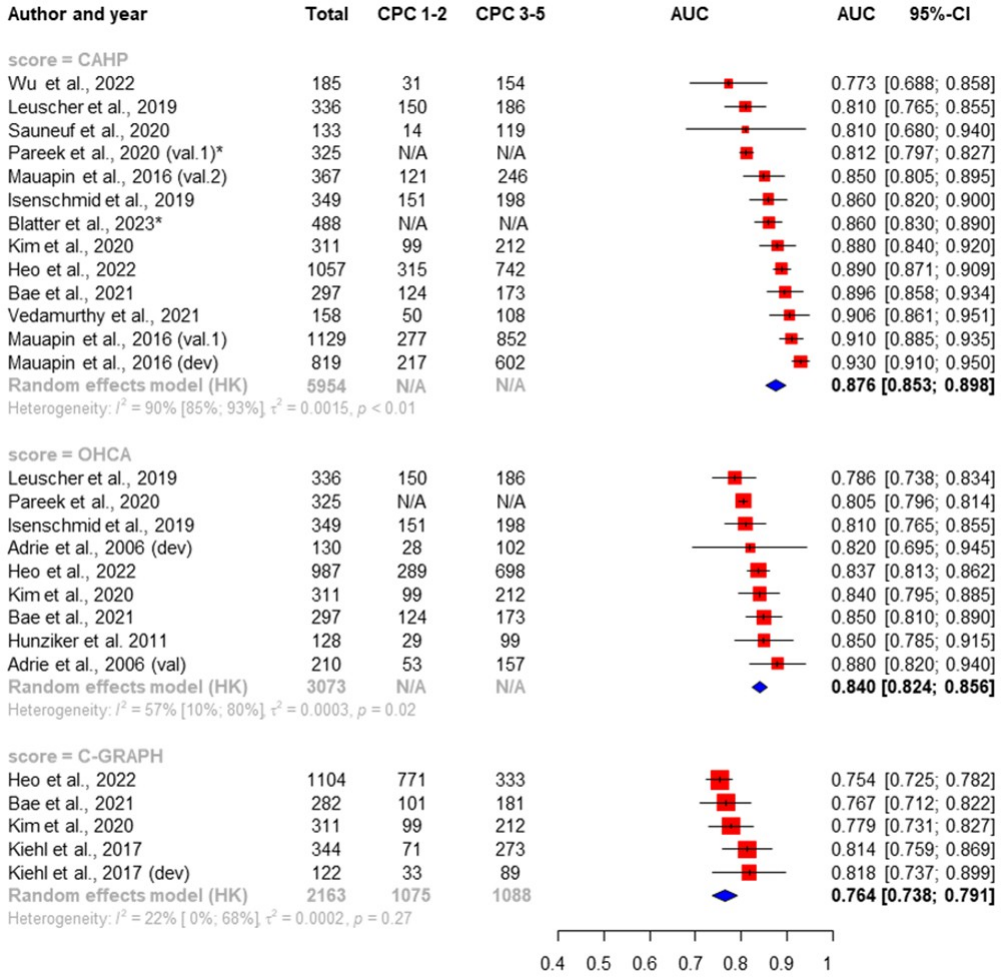
Random-effects pooled AUC of ROC curves and heterogeneity test for CAHP, OHCA and C-GRApH scores predicting neurological outcome at hospital discharge. Red boxes represent the statistical weight that each study contributed to the overall estimate; horizontal black lines represent the 95% CI; blue diamond represent the pooled estimates [26,27,29,38,40,42,47,48,54,57,63,66,72,74]. *AUC and CI was only available.

We identified 14 studies out of 24 which investigated the neurological outcome at hospital discharge [26,27,29,38,40,42,47,48,54,57,63,66,72,74]. Overall, sufficient data were available for three scoring systems (CAHP, C-GRApH, OHCA) to perform a meta-analysis of pooled AUC of ROC curves and to examine heterogeneity. Studies consistently report “poor” (CPC 3–5) or “good” (CPC 1–2) neurological outcome on the CPC scale. Higher AUC value is a more accurate estimate of “poor” neurological outcome by the scoring systems. The highest pooled AUC value was found for the CAHP score (0.876 [0.853–0.898]), and the lowest pooled AUC value was found for the C-GRApH score (0.764 [0.738–0.791]). High heterogeneity was shown in the analysis of CAHP scores (I_2_ = 90%). We found significant difference with a priority in performance of CAHP in comparison with OHCA and C-GRApH scores (CAHP vs. OHCA [p = 0.0046], CAHP vs. C-GRApH [p = 0.0049], OHCA vs. C-GRApH [p = 0.0051]). The results of the analysis are visualized by the forests plots in Fig 2.

### Performance of scores to predict 30-day neurological outcome

**Fig 3:**
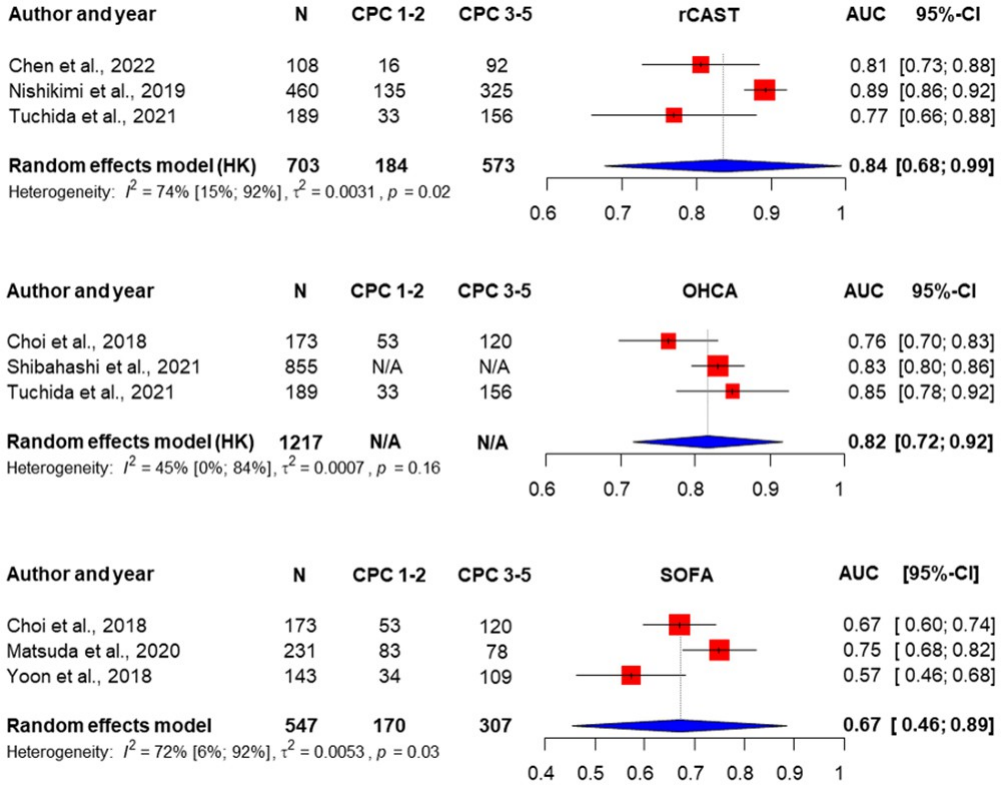
Random-effects pooled AUC of ROC curves and heterogeneity test for rCAST, OHCA and SOFA scores predicting 30-day neurological outcome. Red boxes represent the statistical weight that each study contributed to the overall estimate; horizontal black lines represent the 95% CI; blue diamonds represent the pooled estimates [32,33,56,59,68,71,76].

Of the 24 studies, we identified 7 studies that reported 30-day neurological outcome [32,33,56,59,68,71,76]. There were only 3 scores providing sufficient data for meta-analysis: OHCA, rCAST and SOFA. The highest pooled AUC value was found for the rCAST (0.84 [0.68–0.99]), and the lowest was found for the SOFA score (0.67 [0.46–0.89]). We found no significant difference between pooled AUC values of the scores. The results of the analysis are shown in Fig 3.

### Performance of scores to predict 6-months neurological outcome

**Fig 4:**
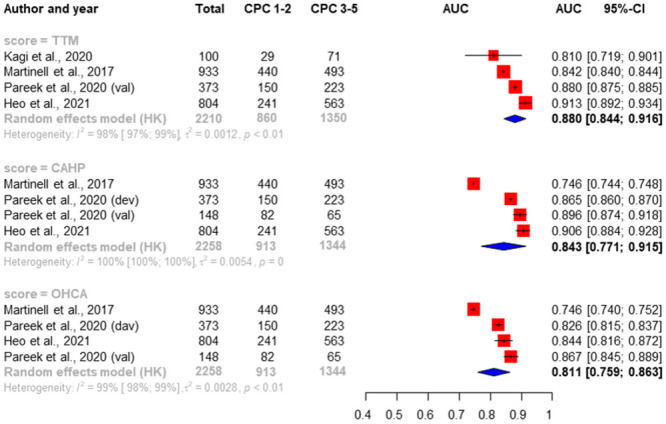
Random-effects pooled AUC of ROC curves and heterogeneity test for TTM, OHCA and CAHP scores predicting 6-month neurological outcome. Red boxes represent the statistical weight that each study contributed to the overall estimate; horizontal black lines represent the 95% CI; blue diamonds represent the pooled estimates [38,46,55,63].

Of the 24 studies, we identified 4 studies that reported 6-months neurological outcome [38,46,55,63]. There were sufficient data for three scoring systems to perform a meta-analysis only for CAHP, OHCA and TTM. The highest pooled AUC value was found for the TTM (0.880 [0.844–0.916]), and the lowest found for the OHCA score (0.811 [0.759–0.863]). High heterogeneity was shown all the analysis (I_2_ = 98–100%). We found significant higher AUC for TTM against OHCA scores (p = 0.0056). The results of the analysis are shown in Fig 4.

### Performance of scores predicting in-hospital mortality

Of the 24 studies, we identified 4 studies that reported in-hospital mortality as a clinical outcome [36,40,42,65]. Only two scoring systems provided satisfactory data to perform a meta-analysis of pooled AUC. We found identical pooled AUC values for the OHCA score (0.84 [0.76–0.92]) and NULL-PLEASE score (0.84 [0.73–0.96]). High heterogeneity was shown shown in the analysis of NULL-PLEASE score (I_2_ = 81%). The results of the analysis are visualized by the forests plots in S2 Fig.

### Performance of SOFA score to predict 30-day mortality outcome

Of the 24 studies, we identified 3 studies that investigated SOFA score with 30-day mortality [33,36,76]. The pooled AUC was 0.71 [0.40–1.02] for the investigated endpoint. High heterogeneity was shown in the analysis (I_2_ = 94%). The results of the analysis are shown in S3 Fig.

### Additional analyses

**Fig 5:**
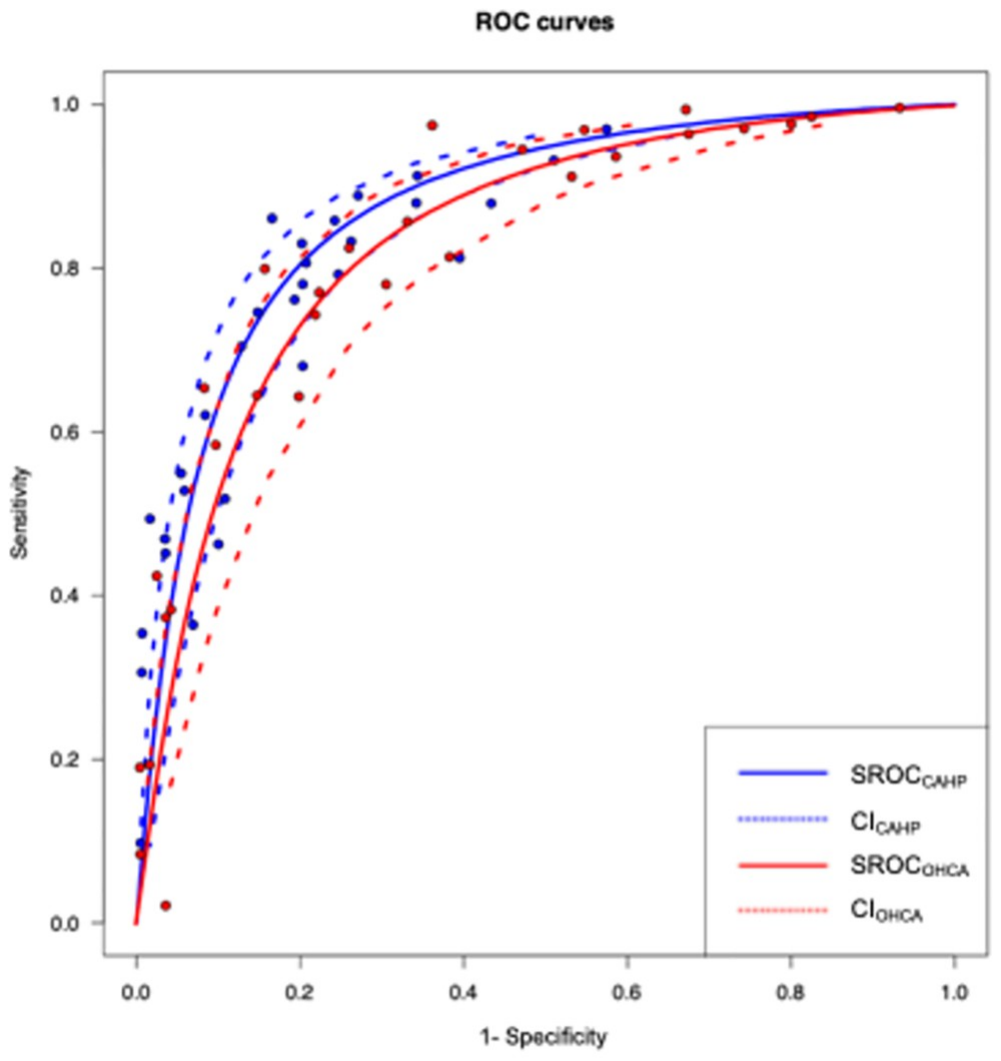
SROC curves along with confidence intervals using the method introduced by Steinhauser and Rücker [20]. The continuous red curve is the SROC curve for the CAHP score system, and the dotted red curve is the corresponding CI. The continuous blue curve is the SROC curve for the OHCA score system, and the dotted blue curve is the corresponding CI. The blue and red dots marks the sensitivity and specificity values given by the individual studies included in the analysis at a fixed threshold.

With the method introduced by Steinhauser and Rücker, we were able to create SROC curves along with CI for two scoring systems (CAHP and OHCA) concerning neurological outcome prediction at hospital discharge [21]. For the other scoring systems, this method was not applicable as there were insufficient sensitivity and specificity values for the different thresholds in the identified manuscripts. The results of the analysis are shown in Fig 5. In addition, we further tested the OHCA and CAHP scores with randomly selected thresholds by repeated analysis 16 times, clearly showing that the CAHP scoring system outperformed the OHCA in the high specificity range (S4 Fig).

**Fig 6:**
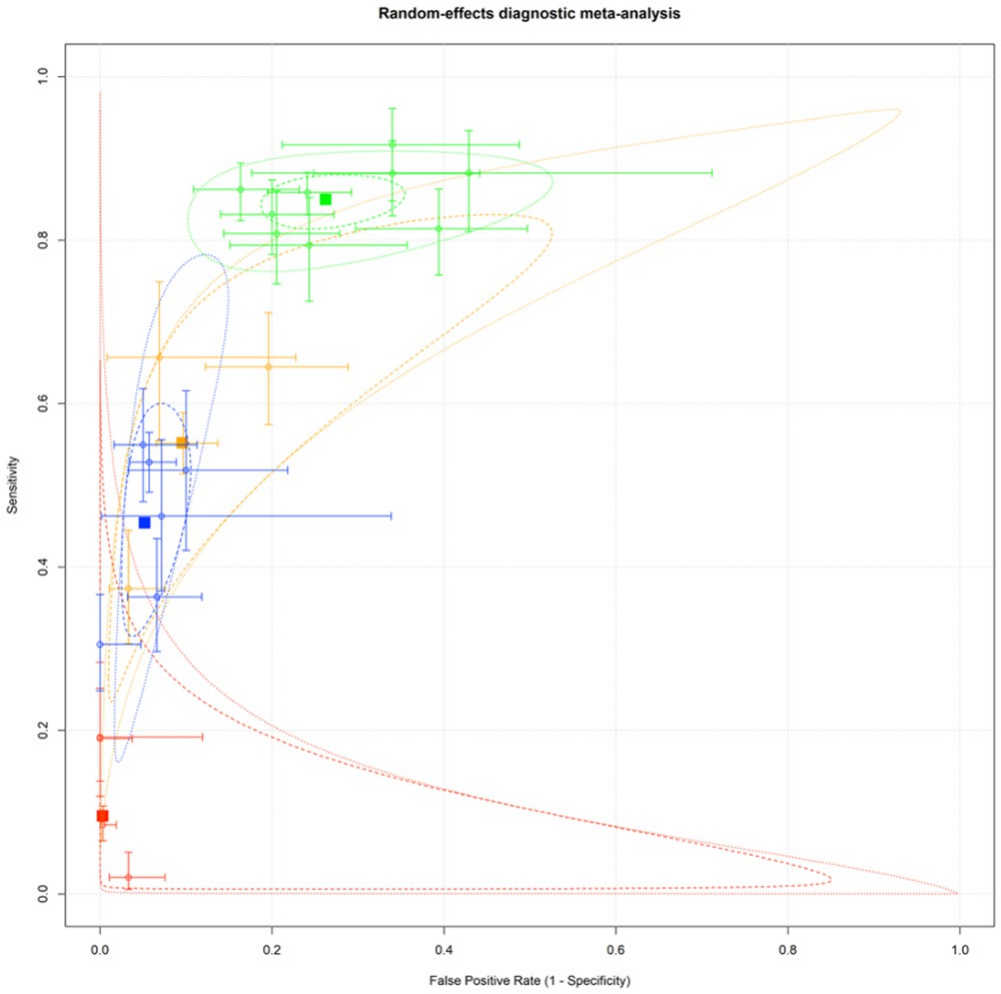
Visualisation of pooled sensitivity and sensitivity analysis of CAHP and OHCA scores at fix threshold. Different colours marks the fixed thresholds: green—CAHP “≥150”; blue–CAHP “≥200”, orange–OHCA “≥40”, red–OHCA “≥60”. The dotted line marks the prediction interval and the dashed line marks the confidence interval of the pooled sensitivity and specificity pair in each fix threshold.

For thresholds 200 and 150 in the case of the CAHP and 60 and 40 in the case of the OHCA, pooled sensitivity and specificity were calculated. In the resulting ROC plot, the pooled sensitivity and specificity of the CAHP when the threshold is 200 are 0.45 (95% CI: [0.38,0.53]) and 0.947 (95% CI [0.924,0.964]). See further details in Fig 6.

## Discussion

Our aim in this study was to review all published predictive scoring systems that estimate the expected outcome in the first few hours after admission, based on simple tests, medical history and resuscitation data. We could confirm our earlier claims for several scoring systems published to predict the prognosis; however, these scores and the reported resuscitated patient populations were very heterogeneous.

The systematic search identified two systematic reviews and one meta-analysis on the topic. The two systematic review listed the prognostic scores or prediction models related to the OHCA and IHCA populations. One of the systematic review made the claim: “A meta-analysis examining the usefulness of scoring systems would be useful, but significant differences between the scores make this currently unfeasible.” Amacher et al. made meta-analysis of C-statistic for only 3 scores, as OHCA, CAHP and GO-FAR scores. Their analysis showed good prognostic accuracy in predicting poor neurological outcome or mortality when using OHCA and CAHP scores, but no discrimination was made according to the time of the endpoints [77–79].

In the systematic review section, we summarized all the available clinical outcome prognostic scoring systems for the successfully resuscitated, adult OHCA patients. Our focus was pointed to those parameters that are easily available at admission to the ICU to be used as most common predictors to create new scoring systems, to refine existing ones, and to give immediate guidance to post-admission patient management. However, the scoring systems we identified used a disseminated range of predictors. It is important to point out that more than 60% of the publications identified in the systematic review were based on patient data before the TTM trial was published, which brought a major paradigm shift in post-resuscitation care [80].

One of the main results of our investigation was that we could prove significant difference between scores predicting the neurological outcome at hospital discharge (CAHP, C-GRApH, OHCA). With additional SROC analyses, we were able to confirm our results based on pooled AUC values. According to the pooled AUC values, the performance of the CAHP and OHCA scores can be interpreted as “good” based on the pre-defined categories. An important finding is the superiority of the CAHP score over the OHCA score (especially in the high specificity range) based on sensitivity and specificity analysis. By using the CAHP scoring system, we can obtain the most accurate information about the neurologic prognosis that will support the decision making on WLST too early.

Based on pooled AUC values the performance of rCAST and OHCA scores to predict 30-day neurological outcome, and the performance of TTM, CAHP and OHCA scores to predict 6-month neurological outcome can be interpreted as “good”. The data available were insufficient for further, more sophisticated analysis.

For the two scoring systems with in-hospital mortality endpoints (OHCA, and NULL-PLEASE), we judged the pooled AUC values to be identical “good”. We did not have sufficient data for further SROC analysis. The SOFA score underperformed the former scores above based on both population size and pooled AUC. Still these data available were insufficient for further analysis.

Based on our meta-analysis results covering the sensitivity, specificity and real-life easiness-to-use characteristics of the score systems a possible subjective, but not mandatory ranking list can be proposed. We ranked the scores by prediction of neurological outcome at hospital discharge (C-GRApH, CAHP, OHCA), the ranking of diagnostic performance was based on the pooled AUC values. Overall, the most useful scoring system was the CAHP, the second was the C-GRApH and the third was OHCA (S5 Table). CAHP and C-GRAPH were ranked the best based on predictor availability, as predictors that were already available during prehospital care or practically immediately after the hospital admission were used. In the case of OHCA, a laboratory-based predictor was also required, so more time was needed to make all the predictors available. C-GRAPH was ranked first in the calculation-based ranking, as the scoring system was based on simple dichotomous predictors. The second was the CAHP, which could be calculated on a sliding scale based on the knowledge of the predictors. The third was the OHCA, which could be calculated using a logarithm-based formula.

### Strengths and limitations

In terms of strengths of our analysis, we followed our protocol, which was registered in advance. To achieve objectivity, multiple analyses were performed, and rigorous methodology was applied. No such comparison and ranking between these highly important scoring systems had been done before.

Given the limitations of this work, many of the analysed articles were retrospective cohorts. Limited data that could be included to our diagnostic meta-analysis, however one of the first comparing analysis among these scoring systems. Due to the diversity of both the identified scoring systems and the clinical endpoints, we included only a few scoring systems in our meta-analysis.

A prediction score produced in a setting may not perform well in another, the risk scores may not give the same predictive accuracy in different populations. This is an important limitation of this study, which issue needs to be described and investigated in more detail in future studies. This systematic review and meta-analysis did not provide enough consistent raw data for the detailed comparisons.

### Implications for practice and research

It is very important to critically assess which part of the results can be immediately implemented into everyday patient care [81,82]. Prognostic scores are expected to provide an objective and accurate estimation of the outcome that can help the clinician to obtain non-emotive reproducible extra information, to depict objectively and prepare relatives for possible undesirable outcomes. Clinical practice suggests that until TTM is completed or targeted diagnostic imaging modalities (CT, MRI) are performed beside the clinical and laboratory evaluation, we cannot be objective enough about the prognosis.

It is important to emphasize that it is completely unethical to make a decision based only on the result of a prediction score that in turn is based on early admission parameters. Consecutive, real-life, well documented patient population should be used to validate all the score systems. In addition to ROC analysis, sensitivity and specificity analyses are also necessary to further reduce unnecessary WTLS. In the future, it would be important to conduct studies on combining biomarkers (e.g. NSE, NfL) with these score systems, and how they affect the predictive accuracy and discriminatory. In this context, some studies identified in the systematic review reported promising results [41,54,70].

The studies included and the scoring system used in this study represented the well-known fact that in some areas of the world (Africa, and South-America) our knowledge and data on sudden cardiac death, resuscitation and post-resuscitation intensive care are very poor and should be given greater emphasis.

## Conclusion

Based on our results CAHP is the most accurate scoring system for predicting the neurological outcome at hospital discharge and is a bit less accurate than TTM score for the 6-month outcome. We recommend the use of the CAHP scoring system in everyday clinical practice not only because of its accuracy and the best performance concerning specificity but also because of the rapid and easy availability of the necessary clinical data for the calculation. The OHCA showed consistently good performance as well to predict neurological outcome at the hospital discharge and at 6-month after the cardiac arrest. Consecutive data collection based real-life registries with a rigorous, reproducible methodology are warranted to compare and validate the outcome prediction scores for cardiac arrest population in the future.

## Supporting information

S1 Fig Graphical presentation of the risk of bias (ROB) and applicability according to PROBAST [15]. (TIF)

S2 Fig Random-effects pooled AUC of ROC curves and heterogeneity test for OHCA and NULL-PLEASE scores predicting in-hospital mortality. Red boxes represent the statistical weight that each study contributed to the overall estimate; horizontal black lines represent the 95% confidence interval; blue diamonds represent the pooled estimates [36,40,42,65].
(TIF)

S3 Fig Random-effects pooled AUC of ROC curves and heterogeneity test for SOFA score predicting 30-day mortality. Red boxes represent the statistical weight that each study contributed to the overall estimate; horizontal black lines represent the 95% confidence interval; blue diamonds represent the pooled estimates [33,36,76].
(TIF)

S4 Fig SROC curves using the non-Bayesian version of the approach introduced by Rutter and Gatsonis with randomly chosen thresholds. (TIF)

S1 Table PRISMA (Preferred Reporting Items for Systematic Reviews and Meta-Analyses) checklist. (DOCX)

S2 Table Available risk scores to predict neurological outcome and mortality following out-of-hospital cardiac arrest. (DOCX)

S3 Table Parameters used by different studies for setting up a prediction model for neurological outcome and mortality. (DOCX)

S4 Table Study quality assessment using Prediction model Risk Of Bias ASsessment Tool (PROBAST). Assessment of risk of bias using Prediction model Risk Of Bias Assessment Tool (PROBAST): “+” indicates low ROB/low concern regarding applicability; “−”indicates high ROB/high concern regarding applicability; and “?” indicates unclear ROB/unclear concern regarding applicability [15,26–76].
(DOCX)

S5 Table Statistical, practical and overall performance of neurological prediction scores (at hospital discharge). (DOCX)

S1 File Searching strategy. (DOCX)
