# Supplementary figures and images for: Prediction performance of scoring systems after out-of-hospital cardiac arrest: A systematic review and meta-analysis
Source: PLoS One. 2024 Feb 1;19(2):e0293704. doi: 10.1371/journal.pone.0293704 (PMC10833585; doi:10.1371/journal.pone.0293704)

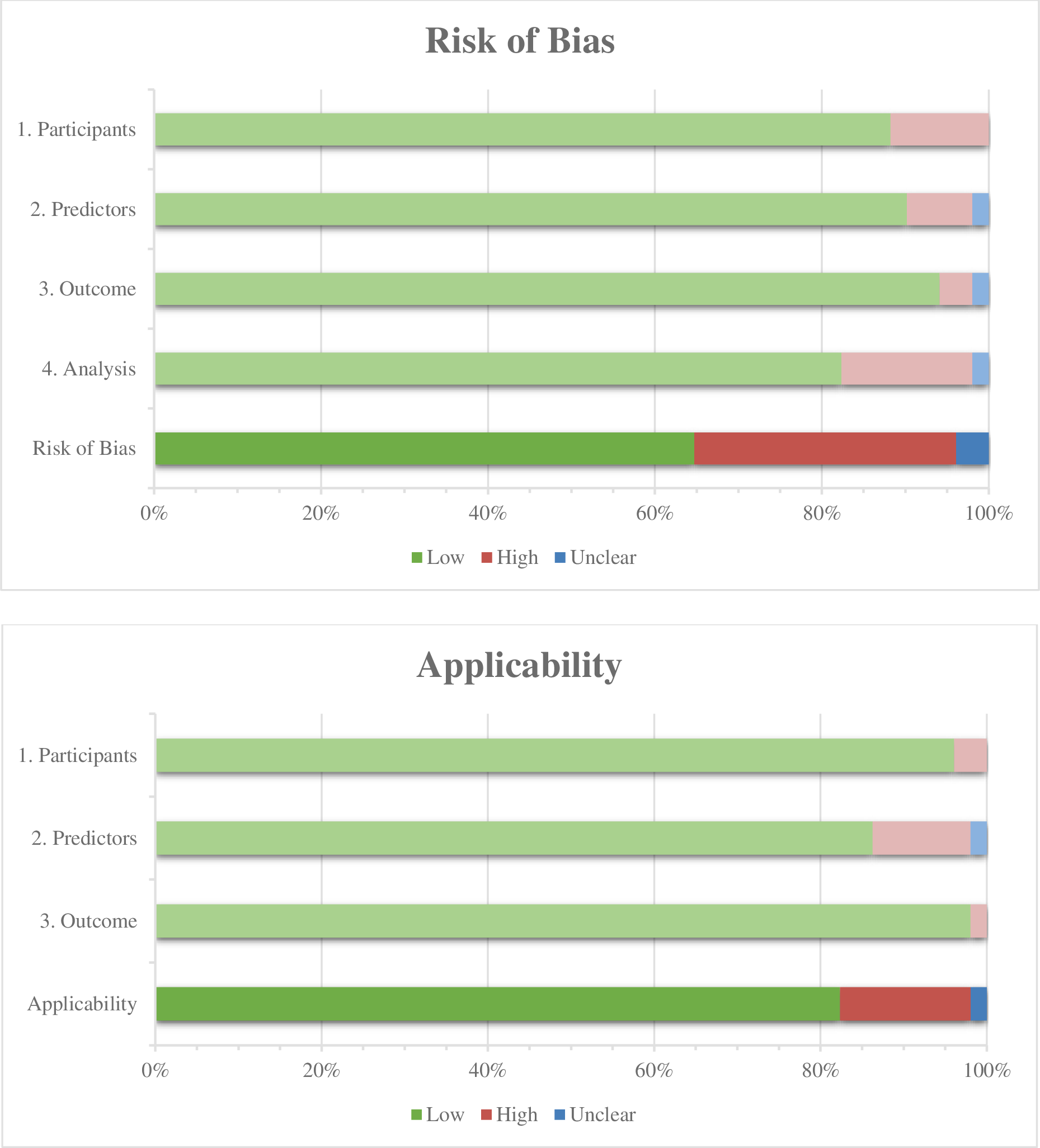

Supplement: S1 Fig — (TIF) [file pone.0293704.s001.tif]

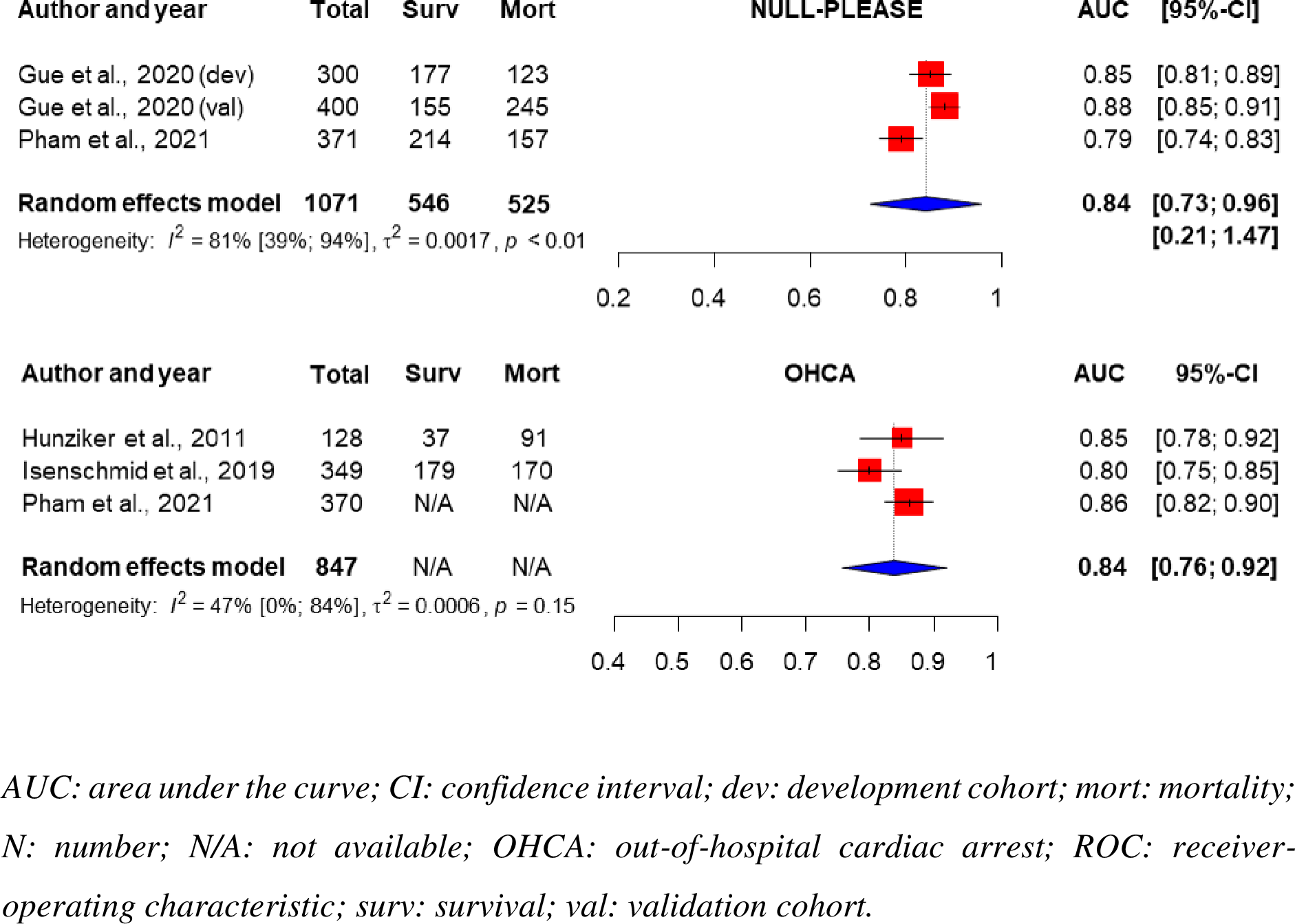

Supplement: S2 Fig — Red boxes represent the statistical weight that each study contributed to the overall estimate; horizontal black lines represent the 95% confidence interval; blue diamonds represent the pooled estimates [36,40,42,65]. (TIF) [file pone.0293704.s002.tif]

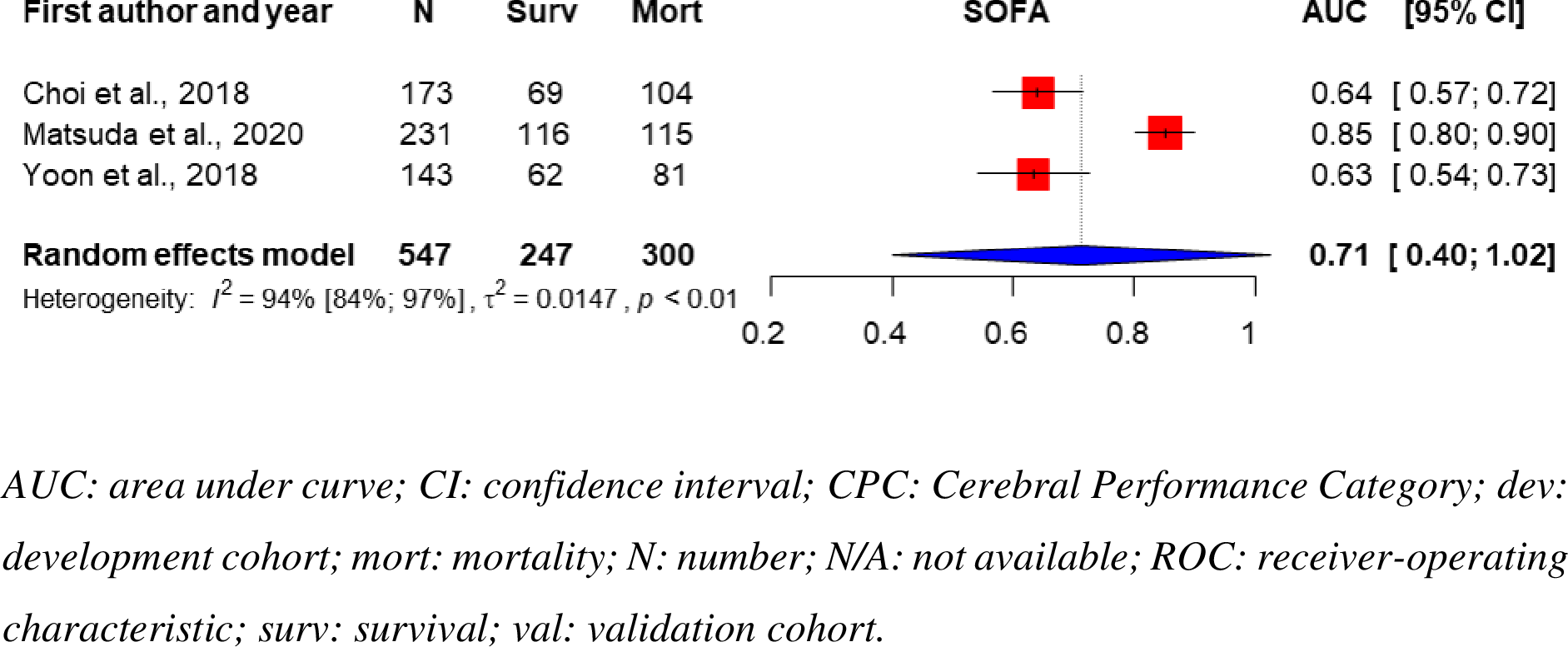

Supplement: S3 Fig — Red boxes represent the statistical weight that each study contributed to the overall estimate; horizontal black lines represent the 95% confidence interval; blue diamonds represent the pooled estimates [33,36,76]. (TIF) [file pone.0293704.s003.tif]

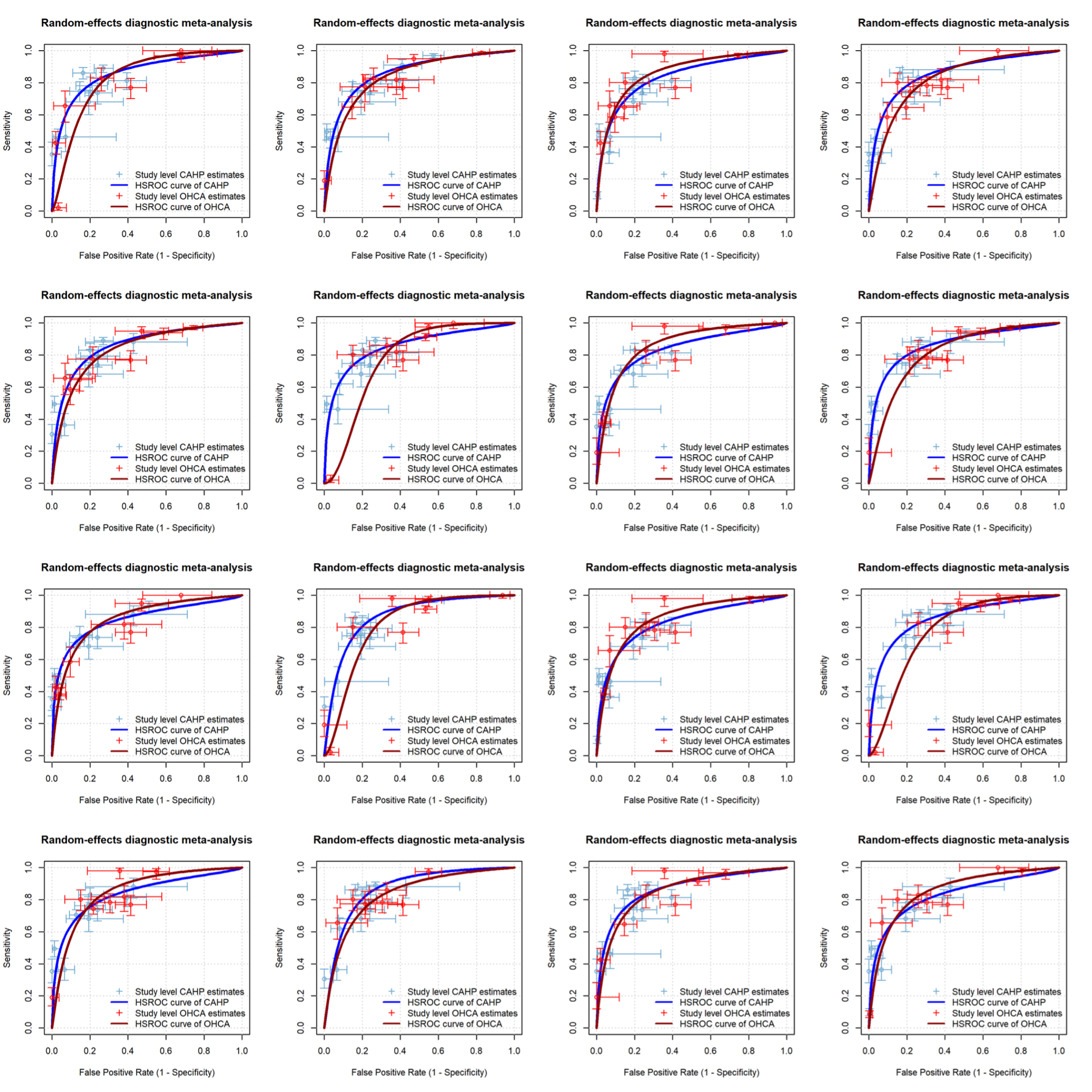

Supplement: S4 Fig — (TIF) [file pone.0293704.s004.tif]
