# Supplementary material for: Prediction performance of scoring systems after out-of-hospital cardiac arrest: A systematic review and meta-analysis
Source: PLoS One. 2024 Feb 1;19(2):e0293704. doi: 10.1371/journal.pone.0293704 (PMC10833585; doi:10.1371/journal.pone.0293704)
Supplement: S2 Table — (DOCX) [file pone.0293704.s006.docx]

**S2 Table. Available risk scores to predict neurological outcome and mortality following out-of-hospital cardiac arrest [26-76].**

| **First author, year** | **Study design** | **Study period** | **Country** | **No. of patients  (% male)** | **No. of TTM patients (%)** | **Neurological outcome** | **Mortality outcome** | **Prediction model  or score system** |
| --- | --- | --- | --- | --- | --- | --- | --- | --- |
| Adrie et al., (26) 2006 | prospective | 1999-2003; 2003-2005 | France | 130^†^ (72); 210^††^ (80) | 14^†^(11); 74^††^(35) | neurological outcome at discharge | - | OHCA |
| Bae et al., (27) 2021 | retrospective | 2014-2016; 2017-2018 | Republic of Korea | 671^†^ (68); 311^††^ (68) | 347^†^ (52);  141^††^(45) | neurological outcome at discharge | - | CAHP, OHCA, PROLOGUE |
| Beom et al., (28) 2021 | prospective | 2015-2018 | Republic of Korea | 496^†^ (83); 227^††^ (86) | 132^†^(27); 44^††^(19) | neurological outcome at discharge | in-hospital mortality | prediction model |
| Blatter et al., (29) 2023 | prospective | 2012-2022 | Switzerland | 687 (72) | 351 (51) | neurological outcome at discharge | - | CAHP, OHCA, PROLOGUE |
| Byrne et al., (30) 2022 | retrospective | 2001-2019 | Denmark | 3881 (77) | N/A | - | 1-day mortality, 30-day mortality | NULL-PLEASE |
| Chen et al., (31) 2021 | retrospective | 2011-2018 | USA | 305 (75) | 305 (100) | neurological outcome at discharge | in-hospital mortality | SLANT |
| Chen et al., (32) 2022 | retrospective | 2015-2021 | Taiwan | 108 (61) | 108 (100) | 28-day neurological outcome | 28-day mortality | rCAST |
| Choi et al., (33) 2018 | retrospective | 2010-2013 | Republic of Korea | 173 (68) | 173 (100) | 30-day neurological outcome | 30-day mortality | APACHE II, OHCA, SAPS II, SOFA, |
| Coppler et al., (34) 2015 | prospective; retrospective | 2011-2013 | USA | 607 (60) | 420 (69) | neurological outcome at discharge | in-hospital mortality | PCAC |
| Dragancea et al., (35) 2015 | RCT | 2010-2013 | Australia, Europe ^†††^ | 312 (81) | 312 (100) | 6-month neurological outcome | - | GCS_M |
| Gue et al., (36) 2020 | prospective; retrospective | 2015-2018 | UK | 300^†^ (88); 400^††^ (75) | N/A | - | in-hospital mortality | NULL-PLEASE |
| Hayakawa et al., (37) 2011 | prospective | 2005-2007 | Japan | 862^†^ (65);  635^††^ (63) | N/A | 1-month neurological outcome | - | prediction model |
| Heo et al., (38) 2022 | prospective | 2015-2018 | Republic of Korea | 1163 (71) | 1163 (100) | 6-month neurological outcome | - | 5-R, CAHP, C-GRApH, CRASS, NULL-PLEASE, OHCA, PHR-RS, PROLOGUE, rCAST, SR-QOLI, TTM |
| Hifumi et al., (39) 2015 | prospective | 2005-2009 | Japan | 302 (81) | 302 (100) | 3-month neurological outcome | - | GCS_M |
| Hunziker et al., (40) 2011 | retrospective | 2006-2008 | USA | 128 (59) | 44 (34) | neurological outcome at discharge | in-hospital mortality | OHCA |
| Hunziker et al., (41) 2021 | prospective | 2012-2016 | Switzerland | 164 (71) | 109 (66) | neurological outcome at discharge | 30-day mortality after discharge | CAHP, OHCA + NfL |
| Isenschmid et al., (42) 2019 | prospective | 2012-2017 | Switzerland | 349 (73) | 200 (57) | neurological outcome at discharge | in-hospital and 30-day mortality | CAHP, OHCA |
| Ishikawa et al., (43) 2013 | retrospective | 2008-2011 | Japan | 750 (60) | 28 (4) | 1-month neurological outcome | 1-month mortality | simple prognostication score |
| Ji et al., (44) 2021 | retrospective | 2014-2015 | UK | 17528† (69); 17078†† (68) | N/A | - | in hospital mortality | perdiction models (Model S1, S2, S3) |
| Jones et al., (45) 2021 | prospective | 2014-2018 | UK | 211 (76) | N/A | - | CED, NED | CREST |
| Kägi et al., (46) 2020 | retrospective | 2016 | Switzerland | 100 (N/A) | N/A | 6-month neurological outcome | - | TTM |
| Kiehl et al., (47) 2017 | prospective cohort | 2008-2012; 2012-2014 | USA | 122^†^ (68);  344^††^ (56) | 122^†^(100); 344^††^(100) | neurological outcome at discharge | in-hospital mortality | C-GRApH |
| Kim et al., (48) 2020 | retrospective | 2009-2017 | Republic of Korea | 311 (71) | 311 (100) | neurological outcome at discharge | - | CAHP, C-GRApH, OHCA |
| Kim et al., (49) 2018 | retrospective | 2011-2016 | Republic of Korea | 143 (66) | 143 (100) | 28-day neurological outcome | - | APACHE II |
| Koltowski et al., (50) 2021 | prospective | 2012-2016 | Poland | 376 (80) | 376 (100) | - | in-hospital mortality | PHR-RS |
| Lim et al., (51) 2021 | prospective | 2016-2020 | Republic of Korea | 4712† (67); 3528†† (63) | 531† (11); 158†† (5) | neurological outcome at discharge | in hospital mortality | ED-PLANN |
| Lin et al., (52) 2022 | retrospective | 2014-2019; 2006-2020; 2006-2010 | Taiwan | 408† (67); 150†† (69); 182†† (70) | 408† (100); 150††(100); 182††(100) | neurological outcome at discharge | - | rCAHP, TIMECARD |
| Liu et al., (53) 2022 | retrospective | 2010-2018 | Singapore | 11404 (66) | N/A | neurological outcome at discharge | - | CRASS |
| Luesher et al., (54) 2019 | prospective cohort | 2012-2017 | Switzerland | 336 (72) | 336 (100) | neurological outcome at discharge | in-hospital mortality | CAHP, OHCA + NSE |
| Martinell et al., (55) 2017 | RCT | 2010-2013 | Australia, Europe ^†††^ | 933 (81) | N/A | 6-month neurological outcome | - | CAHP, OHCA, TTM |
| Matsuda et al., (56) 2020 | retrospective | 2015-2018 | Japan | 231 (78) | 144 (62) | 30-day neurological outcome | 30-day mortality | SOFA |
| Maupain et al., (57) 2016 | prospective | 2011-2012; 2007-2010; 2013-2014 | France | 819^†^ (69); 1129^††^ (67); 367^††^ (71) | N/A | neurological outcome at ICU discharge | - | CAHP |
| Nadolny et al., (58) 2021 | prospective | 2018 | Poland | 218 (65) | 218 (100) | - | in-hospital mortality | GCS |
| Nishikimi et al., (59) 2019 | prospective | 2014-2015 | Japan | 460 (75) | 460 (100) | 30- and 90-day neurological outcome | 30- and 90-day mortality | rCAST |
| Nishioka et al., (60) 2021 | prospective | 2013-2015; 2016-2017 | Japan | 1329† (72); 1025†† (73) | N/A | 90-day neurological outcome | - | models 1 and 2 |
| Oh et al., (61) 2019 | retrospective | 2015-2016 | Republic of Korea | 166 (78) | 46 (28) | neurological outcome at discharge | in hospital mortality | VIS |
| Okada et al., (62) 2012 | retrospective | 2006-2011 | Japan | 66 (62) | 66 (100) | neurological outcome at discharge | - | 5-R |
| Pareek et al., (63) 2020 | prospective | 2012-2017 | UK; Slovenia | 373^†^ (74); 325^††^ (82); 148^††^(76) | N/A | neurological outcome at discharge; 6-month neurological outcome | - | CAHP, MIRACLE 2, OHCA, TTM |
| Paul et al., (64) 2023 | prospective | 2007-2017 | France | 1543 (70) | N/A | - | in hospital mortality | CAHP |
| Pham et al., (65) 2021 | prospective | 2009-2019 | France | 386 (79) | 337 (87) | - | in hospital mortality | CAHP, NULL-PLEASE, OHCA |
| Sauneuf et al., (66) 2020 | retrospective | 2009-2016 | France | 176 (72) | 79 (45) | neurological outcome at discharge | - | CAHP |
| Schriefl et al., (67) 2022 | prospective | 2013-2018 | Austria | 1051 (61) | 738 (70) | 30-day neurological outcome | - | PROLOGUE |
| Shibahashi et al., (68) 2020 | retrospective | 2012-2013 | Japan | 2468 (68) | N/A | 30-day neurological outcome | - | CAHP, sCAHP, OHCA, sOHCA |
| Shih et al., (69) 2019 | prospective | 2015-2017 | China | 852^†^(62); 859^††^(62) | N/A | neurological outcome at discharge | - | SWAP |
| Song et al., (70) 2021 | retrospective | 2018-2020 | Republic of Korea | 106 (74) | 106 (100) | 3-month neurological outcome | - | CAHP, OHCA + NSE |
| Tsuchida et al., (71) 2021 | retrospective | 2015-2018 | Japan | 189 (59) | 100 (53) | 1-month neurological outcome | - | CAHP, CAST, NULL-PLEASE, OHCA, rCAST |
| Vedamurthy et al., (72) 2021 | retrospective | 2016-2019 | USA | 158 (73) | N/A | neurological outcome at discharge | in hospital mortality | CAHP |
| Wang et al., (73) 2019 | prospective | 2011-2017 | Taiwan | 412 (68) | N/A | neurological outcome at discharge | - | CAHP, OHCA |
| Weiss et al., (74) 2015 | prospective | 2009-2011 | France | 85 (72) | N/A | 6-month neurological outcome | 6-month mortality | FOUR, GCS |
| Wu et al., (75) 2022 | retrospective | 2015-2019 | Taiwan | 200 (61) | N/A | neurological outcome at discharge | - | CAHP, MIRACLE 2 |
| Yoon et al., (76) 2018 | retrospective | 2010-2015 | Republic of Korea | 143 (66) | 143 (100) | 28-day neurological outcome | 28-day mortality | APACHE II, SOFA |

^†^: development cohort, ^††^: validation cohort, ^†††^: Denmark, Italy, Luxembourg, Netherlands, Norway, Sweden, Switzerland ,United Kingdom

*APACHE II: Acute Physiology and Chronic Health Evaluation II , CAHP: Cardiac Arrest Hospital Prognosis, CED: circulatory-aetiology deaths, CRASS: CaRdiac Arrest Survival Score, GCS: Glasgow Coma Scale, ICU: intensive care unit, N/A: not available, NED: neurological-aetiology deaths, NfL:* *neurofilament light, No: number, NSE: neuron specific enolase, OHCA: out-of-hospital cardiac arrest, PCAC: Pittsburgh Cardiac Arrest Category, PHR: Polish hypothermia registry,* *PROLOGUE: PROgnostication using LOGistic regression model for Unselected adult cardiac arrest patients in the Early stages, rCAST: revised post-cardiac arrest syndrome for therapeutic hypothermia, RCT: randomized controlled trial, SAPS II: Simplified Acute Physiology Score II, SOFA: Sequential Organ Failure Assessment, TIMECARD: TaIwan network of targeted temperature ManagEment for CARDiac arrest, TTM: targeted temperature management, UK: United Kingdom, USA: United States of America, VIS: vasoactive-inotropic score.*
