# Supplementary material for: Prediction performance of scoring systems after out-of-hospital cardiac arrest: A systematic review and meta-analysis
Source: PLoS One. 2024 Feb 1;19(2):e0293704. doi: 10.1371/journal.pone.0293704 (PMC10833585; doi:10.1371/journal.pone.0293704)
Supplement: S3 Table — (DOCX) [file pone.0293704.s007.docx]

**S3 Table. Parameters used by different studies for setting up a prediction model for neurological outcome and mortality [26-76].**

| Variable | No of scores which using variable | 5-R score | APACHE II | Beom's prediction model | CAHP | CAHP + NfL | CAHP + NSE | C-GRApH | CRASS | CREST | ED-PLANN | FOUR | GCS | GCS_M | Hayakawa's prediction model | Ji's predicton modell | MIRACLE 2 | Nishioka's model | NULL-PLEASE | OHCA | OHCA + NfL | OHCA + NSE | PCAC | PHR-RS | PROLOGUE | rCAST | SAPS II | simple prognostication score | SLANT | SOFA | SWAP | TIMECARD | TTM | VIS |
| --- | --- | --- | --- | --- | --- | --- | --- | --- | --- | --- | --- | --- | --- | --- | --- | --- | --- | --- | --- | --- | --- | --- | --- | --- | --- | --- | --- | --- | --- | --- | --- | --- | --- | --- |
| Initial rhythm | 25 | 1 |  |  | 1 | 1 | 1 | 1 | 1 | 1 | 1 |  |  |  | 1 | 1 | 1 | 1 | 1 | 1 | 1 | 1 | 1 | 1 | 1 | 1 |  | 1 | 1 |  | 1 | 1 | 1 |  |
| Age | 18 |  | 1 | 1 | 1 | 1 | 1 | 1 | 1 |  | 1 |  |  |  | 1 | 1 | 1 | 1 | 1 |  |  |  |  | 1 | 1 |  | 1 |  |  |  | 1 |  | 1 |  |
| No-flow time | 14 | 1 |  |  | 1 | 1 | 1 |  | 1 |  |  |  |  |  |  |  |  | 1 | 1 | 1 | 1 | 1 | 1 | 1 |  |  |  |  |  |  |  | 1 | 1 |  |
| Low-flow time | 13 |  |  |  | 1 | 1 | 1 |  |  |  |  |  |  |  |  |  |  | 1 | 1 | 1 | 1 | 1 | 1 | 1 | 1 |  |  |  |  |  |  | 1 | 1 |  |
| pH | 13 |  | 1 |  | 1 | 1 | 1 | 1 |  |  | 1 |  |  |  |  |  | 1 | 1 | 1 |  |  |  |  |  |  | 1 |  | 1 |  |  | 1 |  | 1 |  |
| Witnessed / unwitnessed arrest | 12 |  |  |  |  |  |  |  | 1 |  |  |  |  |  |  | 1 | 1 | 1 | 1 |  |  |  |  | 1 | 1 | 1 |  | 1 | 1 |  | 1 | 1 |  |  |
| Epinephrine | 10 |  |  |  | 1 | 1 | 1 |  | 1 |  |  |  |  |  |  |  | 1 | 1 |  |  |  |  | 1 |  | 1 |  |  |  | 1 |  |  |  | 1 |  |
| Glasgow Coma Scale | 9 |  | 1 |  |  |  |  |  |  |  |  |  | 1 |  |  |  |  | 1 |  |  |  |  | 1 |  | 1 |  | 1 | 1 |  | 1 |  | 1 |  |  |
| Lactate | 9 |  |  |  |  |  |  |  |  | 1 | 1 |  |  |  |  |  |  | 1 | 1 | 1 | 1 | 1 |  |  | 1 | 1 |  |  |  |  |  |  |  |  |
| Creatinine | 8 |  | 1 |  |  |  |  |  |  |  |  |  |  |  |  |  |  | 1 |  | 1 | 1 | 1 | 1 |  | 1 |  |  |  |  | 1 |  |  |  |  |
| Time to ROSC (no flow + low-flow time) | 7 | 1 |  | 1 |  |  |  |  | 1 | 1 |  |  |  |  | 1 |  |  |  |  |  |  |  |  |  |  | 1 |  |  | 1 |  |  |  |  |  |
| Motor response | 5 |  |  |  |  |  |  |  |  |  |  | 1 |  | 1 |  |  |  |  |  |  |  |  | 1 |  |  | 1 |  |  |  |  |  |  | 1 |  |
| Prehospital ROSC | 5 |  |  | 1 |  |  |  |  |  |  | 1 |  |  |  | 1 |  |  | 1 |  |  |  |  |  |  |  |  |  | 1 |  |  |  |  |  |  |
| Pupillary light or corneal reflex | 5 | 1 |  |  |  |  |  |  |  |  |  |  |  |  |  |  | 1 |  |  |  |  |  |  |  | 1 |  |  | 1 |  |  |  |  | 1 |  |
| Arrest setting | 4 |  |  |  | 1 | 1 | 1 |  | 1 |  |  |  |  |  |  |  |  |  |  |  |  |  |  |  |  |  |  |  |  |  |  |  |  |  |
| Bilirubin | 3 |  |  |  |  |  |  |  |  |  |  |  |  |  |  |  |  |  |  |  |  |  | 1 |  |  |  | 1 |  |  | 1 |  |  |  |  |
| Etiology | 3 |  |  |  |  |  |  |  | 1 |  |  |  |  |  |  | 1 |  |  |  |  |  |  |  |  |  |  |  | 1 |  |  |  |  |  |  |
| FiO₂ | 3 |  | 1 |  |  |  |  |  |  |  |  |  |  |  |  |  |  |  |  |  |  |  | 1 |  |  |  |  |  |  | 1 |  |  |  |  |
| Mean arterial pressure | 3 |  | 1 |  |  |  |  |  |  |  |  |  |  |  |  |  |  |  |  |  |  |  | 1 |  |  |  |  |  |  | 1 |  |  |  |  |
| Ongoing CPR on arrival | 3 |  |  |  |  |  |  |  | 1 |  |  |  |  |  |  |  |  | 1 | 1 |  |  |  |  |  |  |  |  |  |  |  |  |  |  |  |
| Potassium | 3 |  | 1 |  |  |  |  |  |  |  |  |  |  |  |  |  |  |  |  |  |  |  |  |  | 1 |  | 1 |  |  |  |  |  |  |  |
| Sodium | 3 |  | 1 |  |  |  |  |  |  |  |  |  |  |  |  |  |  |  |  |  |  |  |  |  |  |  | 1 | 1 |  |  |  |  |  |  |
| White blood cell count | 3 |  | 1 |  |  |  |  |  |  |  |  |  |  |  |  |  |  |  |  |  |  |  |  |  |  |  | 1 |  | 1 |  |  |  |  |  |
| Brainstem reflexes | 2 |  |  |  |  |  |  |  |  |  |  | 1 |  |  |  |  |  |  |  |  |  |  | 1 |  |  |  |  |  |  |  |  |  |  |  |
| Changing rhythm | 2 |  |  |  |  |  |  |  |  |  |  |  |  |  | 1 |  | 1 |  |  |  |  |  |  |  |  |  |  |  |  |  |  |  |  |  |
| Chronic disease | 2 |  |  |  |  |  |  |  | 1 |  |  |  |  |  |  |  |  |  |  |  |  |  |  |  |  |  | 1 |  |  |  |  |  |  |  |
| Coronary artery disease, known pre-arrest | 2 |  |  |  |  |  |  | 1 |  | 1 |  |  |  |  |  |  |  |  |  |  |  |  |  |  |  |  |  |  |  |  |  |  |  |  |
| Eye response | 2 |  |  |  |  |  |  |  |  |  |  | 1 |  |  |  |  |  |  |  |  |  |  | 1 |  |  |  |  |  |  |  |  |  |  |  |
| Gender | 2 |  |  |  |  |  |  |  |  |  |  |  |  |  |  | 1 |  | 1 |  |  |  |  |  |  |  |  |  |  |  |  |  |  |  |  |
| Heart rate/pulse | 2 |  | 1 |  |  |  |  |  |  |  |  |  |  |  |  |  |  |  |  |  |  |  |  |  |  |  | 1 |  |  |  |  |  |  |  |
| In hospital re-arrest | 2 | 1 |  |  |  |  |  |  |  |  |  |  |  |  |  |  |  |  |  |  |  |  |  | 1 |  |  |  |  |  |  |  |  |  |  |
| NfL | 2 |  |  |  |  | 1 |  |  |  |  |  |  |  |  |  |  |  |  |  |  | 1 |  |  |  |  |  |  |  |  |  |  |  |  |  |
| NSE | 2 |  |  |  |  |  | 1 |  |  |  |  |  |  |  |  |  |  |  |  |  |  | 1 |  |  |  |  |  |  |  |  |  |  |  |  |
| PaCO₂ | 2 |  |  |  |  |  |  |  |  |  |  |  |  |  |  |  |  | 1 |  |  |  |  |  |  |  |  |  |  |  |  |  |  | 1 |  |
| PaO₂ | 2 |  |  |  |  |  |  |  |  |  |  |  |  |  |  |  |  |  |  |  |  |  | 1 |  |  |  |  |  |  | 1 |  |  |  |  |
| PaO₂/FiO₂ ratio | 2 |  |  |  |  |  |  |  |  |  |  |  |  |  |  |  |  |  |  |  |  |  | 1 |  |  |  | 1 |  |  |  |  |  |  |  |
| Platelets, ×10³/µL | 2 |  |  |  |  |  |  |  |  |  |  |  |  |  |  |  |  |  |  |  |  |  | 1 |  |  |  |  |  |  | 1 |  |  |  |  |
| Respiration pattern | 2 |  |  |  |  |  |  |  |  |  |  | 1 |  |  |  |  |  |  |  |  |  |  | 1 |  |  |  |  |  |  |  |  |  |  |  |
| Serum glucose | 2 |  |  |  |  |  |  | 1 |  |  |  |  |  |  |  |  |  | 1 |  |  |  |  |  |  |  |  |  |  |  |  |  |  |  |  |
| Temperature | 2 |  | 1 |  |  |  |  |  |  |  |  |  |  |  |  |  |  |  |  |  |  |  |  |  |  |  | 1 |  |  |  |  |  |  |  |
| Acute renal failure | 1 |  | 1 |  |  |  |  |  |  |  |  |  |  |  |  |  |  |  |  |  |  |  |  |  |  |  |  |  |  |  |  |  |  |  |
| Advanced airway management during cardiac arrest | 1 |  |  |  |  |  |  |  |  |  |  |  |  |  |  |  |  | 1 |  |  |  |  |  |  |  |  |  |  |  |  |  |  |  |  |
| Amiodarone | 1 |  |  |  |  |  |  |  | 1 |  |  |  |  |  |  |  |  |  |  |  |  |  |  |  |  |  |  |  |  |  |  |  |  |  |
| Bicarbonate | 1 |  |  |  |  |  |  |  |  |  |  |  |  |  |  |  |  |  |  |  |  |  |  |  |  |  | 1 |  |  |  |  |  |  |  |
| Bystander AED use | 1 |  |  |  |  |  |  |  |  |  |  |  |  |  |  |  |  | 1 |  |  |  |  |  |  |  |  |  |  |  |  |  |  |  |  |
| Bystander-initiated CPR | 1 |  |  |  |  |  |  |  |  |  |  |  |  |  |  |  |  |  |  |  |  |  |  |  |  |  |  | 1 |  |  |  |  |  |  |
| Cardiogenic shock | 1 |  |  |  |  |  |  |  |  | 1 |  |  |  |  |  |  |  |  |  |  |  |  |  |  |  |  |  |  |  |  |  |  |  |  |
| CRP | 1 |  |  |  |  |  |  |  |  |  |  |  |  |  |  |  |  |  |  |  |  |  |  |  |  |  |  | 1 |  |  |  |  |  |  |
| Dose of vasoactive or inotropic agents | 1 |  |  |  |  |  |  |  |  |  |  |  |  |  |  |  |  |  |  |  |  |  |  |  |  |  |  |  |  |  |  |  |  | 1 |
| Ejection fraction < 30% | 1 |  |  |  |  |  |  |  |  | 1 |  |  |  |  |  |  |  |  |  |  |  |  |  |  |  |  |  |  |  |  |  |  |  |  |
| End-stage chronic kidney disease on dialysis | 1 |  |  |  |  |  |  |  |  |  |  |  |  |  |  |  |  |  | 1 |  |  |  |  |  |  |  |  |  |  |  |  |  |  |  |
| Extracardiac origin (eg. trauma, pulmonary embolism) | 1 |  |  |  |  |  |  |  |  |  |  |  |  |  |  |  |  |  | 1 |  |  |  |  |  |  |  |  |  |  |  |  |  |  |  |
| Hematocrit | 1 |  | 1 |  |  |  |  |  |  |  |  |  |  |  |  |  |  |  |  |  |  |  |  |  |  |  |  |  |  |  |  |  |  |  |
| Hemoglobin | 1 |  |  |  |  |  |  |  |  |  |  |  |  |  |  |  |  |  |  |  |  |  |  |  | 1 |  |  |  |  |  |  |  |  |  |
| History of severe organ failure or immunocompromise | 1 |  | 1 |  |  |  |  |  |  |  |  |  |  |  |  |  |  |  |  |  |  |  |  |  |  |  |  |  |  |  |  |  |  |  |
| Malignancy | 1 |  |  |  |  |  |  |  |  |  |  |  |  |  |  |  |  |  |  |  |  |  |  |  |  |  |  |  |  |  |  | 1 |  |  |
| Mechanical CPR | 1 |  |  |  |  |  |  |  | 1 |  |  |  |  |  |  |  |  |  |  |  |  |  |  |  |  |  |  |  |  |  |  |  |  |  |
| No emergent CAG | 1 |  |  |  |  |  |  |  |  |  |  |  |  |  |  |  |  |  |  |  |  |  |  |  |  |  |  |  |  |  |  | 1 |  |  |
| Phosphate | 1 |  |  |  |  |  |  |  |  |  |  |  |  |  |  |  |  |  |  |  |  |  |  |  | 1 |  |  |  |  |  |  |  |  |  |
| Place of cardiac arrest | 1 |  |  |  |  |  |  |  |  |  |  |  |  |  |  |  |  |  |  |  |  |  |  |  |  |  |  |  |  |  |  |  | 1 |  |
| Prehospital defibrillation by EMS providers | 1 |  |  | 1 |  |  |  |  |  |  |  |  |  |  |  |  |  |  |  |  |  |  |  |  |  |  |  |  |  |  |  |  |  |  |
| Respiratory rate | 1 |  | 1 |  |  |  |  |  |  |  |  |  |  |  |  |  |  |  |  |  |  |  |  |  |  |  |  |  |  |  |  |  |  |  |
| Serum albumin | 1 |  |  |  |  |  |  |  |  |  |  |  |  |  |  |  |  | 1 |  |  |  |  |  |  |  |  |  |  |  |  |  |  |  |  |
| Serum urea | 1 |  |  |  |  |  |  |  |  |  |  |  |  |  |  |  |  |  |  |  |  |  |  |  |  |  | 1 |  |  |  |  |  |  |  |
| Sodium bicarbonate used during CPR | 1 |  |  |  |  |  |  |  |  |  |  |  |  |  |  |  |  |  |  |  |  |  |  |  |  |  |  |  |  |  |  | 1 |  |  |
| Systolic blood pressure | 1 |  |  |  |  |  |  |  |  |  |  |  |  |  |  |  |  |  |  |  |  |  |  |  |  |  | 1 |  |  |  |  |  |  |  |
| Type of admission | 1 |  |  |  |  |  |  |  |  |  |  |  |  |  |  |  |  |  |  |  |  |  |  |  |  |  | 1 |  |  |  |  |  |  |  |
| Urine output | 1 |  |  |  |  |  |  |  |  |  |  |  |  |  |  |  |  |  |  |  |  |  |  |  |  |  | 1 |  |  |  |  |  |  |  |
| All parameters per score | - | 5 | 15 | 4 | 7 | 8 | 8 | 5 | 12 | 6 | 5 | 4 | 1 | 1 | 5 | 5 | 7 | 18 | 10 | 5 | 6 | 6 | 16 | 6 | 12 | 6 | 15 | 10 | 5 | 7 | 4 | 8 | 10 | 1 |
| All stuedies per score | - | 1 | 3 | 1 | 11 | 1 | 2 | 2 | 2 | 1 | 1 | 1 | 2 | 2 | 1 | 1 | 1 | 1 | 3 | 12 | 1 | 2 | 1 | 1 | 4 | 2 | 1 | 1 | 1 | 3 | 1 | 1 | 2 | 1 |
| Predicts neurological outcome | - | ✓ | ✓ | ✓ | ✓ | ✓ | ✓ | ✓ | ✓ | 🗴 | ✓ | ✓ | ✓ | ✓ | ✓ | ✓ | ✓ | ✓ | ✓ | ✓ | ✓ | ✓ | 🗴 | ✓ | ✓ | ✓ | ✓ | ✓ | ✓ | ✓ | ✓ | ✓ | ✓ | ✓ |
| Predicts mortality outcome | - | 🗴 | ✓ | ✓ | ✓ | ✓ | ✓ | ✓ | 🗴 | ✓ | ✓ | ✓ | ✓ | 🗴 | 🗴 | 🗴 | ✓ | 🗴 | ✓ | ✓ | ✓ | ✓ | ✓ | ✓ | 🗴 | ✓ | ✓ | ✓ | ✓ | ✓ | 🗴 | 🗴 | 🗴 | ✓ |

*CAD: coronary artery disease; CPR: cardiopulmonary resuscitation; CRP: C-reactive protein; FiO_2_: fraction of inspired oxygen; NfL: neurofilament light chain; NSE: neuron-specific enolase; PaCO_2_: partial pressure of carbon-dioxide; PaO_2_: partial pressure of oxygen; ROSC :return of spontaneous circulation.*
