# Supplementary material for: Prediction performance of scoring systems after out-of-hospital cardiac arrest: A systematic review and meta-analysis
Source: PLoS One. 2024 Feb 1;19(2):e0293704. doi: 10.1371/journal.pone.0293704 (PMC10833585; doi:10.1371/journal.pone.0293704)
Supplement: S4 Table — Assessment of risk of bias using Prediction model Risk Of Bias Assessment Tool (PROBAST): “+” indicates low ROB/low concern regarding applicability; “−”indicates high ROB/high concern regarding applicability; and “?” indicates unclear ROB/unclear concern regarding applicability [15,26–76]. (DOCX) [file pone.0293704.s008.docx]

**S4 Table.** **Assessment of risk of bias using Prediction model Risk Of Bias Assessment Tool (PROBAST).** “+” indicates low ROB/low concern regarding applicability; “−“ indicates high ROB/high concern regarding applicability; and “?” indicates unclear ROB/unclear concern regarding applicability [15, 26-76].

| **First author, year** | **ROB** | | | | **Applicability** | | | **Overall** | |
| --- | --- | --- | --- | --- | --- | --- | --- | --- | --- |
|  | Participants | Predictors | Outcome | Analysis | Participants | Predictors | Outcome | ROB | Applicability |
| Adrie et al., (26) 2006 | + | + | + | + | + | + | + | + | + |
| Bae et al., (27) 2021 | + | + | + | - | + | + | + | - | + |
| Beom et al., (28) 2021 | + | + | + | + | + | + | + | + | + |
| Blatter et al., (29) 2023 | + | + | + | + | + | + | + | + | + |
| Byrne et al., (30) 2022 | + | + | - | - | + | + | + | - | + |
| Chen et al., (31) 2021 | + | + | + | + | + | + | + | + | + |
| Chen et al., (32) 2022 | + | + | + | - | + | + | + | - | + |
| Choi et al., (33) 2018 | + | + | + | + | + | + | + | + | + |
| Coppler et al., (34) 2015 | + | + | + | + | + | ? | + | + | ? |
| Dragancea et al., (35) 2015 | + | + | + | + | + | + | + | + | + |
| Gue et al., (36) 2020 | + | + | + | + | + | + | + | + | + |
| Hayakawa et al., (37) 2011 | + | + | + | + | + | + | + | + | + |
| Heo et al., (38) 2022 | + | + | + | + | + | + | + | + | + |
| Hifumi et al., (39) 2015 | + | + | + | + | + | + | + | + | + |
| Hunziker et al., (40) 2011 | + | + | + | + | + | + | + | + | + |
| Hunziker et al., (41) 2021 | + | ? | + | + | + | - | + | ? | - |
| Isenschmid et al., (42) 2019 | + | + | + | + | + | + | + | + | + |
| Ishikawa et al., (43) 2013 | + | + | + | + | + | + | + | + | + |
| Ji et al., (44) 2021 | + | + | + | - | + | + | + | - | + |
| Jones et al., (45) 2021 | - | + | - | + | + | - | - | - | - |
| Kägi et al., (46) 2020 | - | - | ? | - | + | - | + | - | - |
| Kiehl et al., (47) 2017 | + | + | + | + | + | + | + | + | + |
| Kim et al., (48) 2020 | + | - | + | + | + | - | + | - | - |
| Kim et al., (49) 2018 | + | + | + | + | + | + | + | + | + |
| Koltowski et al., (50) 2021 | + | + | + | + | + | + | + | + | + |
| Lim et al., (51) 2021 | + | + | + | - | + | + | + | - | + |
| Lin et al., (52) 2022 | + | + | + | - | + | + | + | - | + |
| Liu et al., (53) 2022 | + | + | + | + | + | + | + | + | + |
| Luesher et al., (54) 2019 | + | - | + | + | + | - | + | - | - |
| Martinell et al., (55) 2017 | + | + | + | + | + | + | + | + | + |
| Matsuda et al., (56) 2020 | + | + | + | + | + | + | + | + | + |
| Maupain et al., (57) 2016 | + | + | + | ? | + | + | + | ? | + |
| Nadolny et al., (58) 2021 | + | + | + | + | + | + | + | + | + |
| Nishikimi et al., (59) 2019 | - | + | + | + | - | + | + | - | - |
| Nishioka et al., (60) 2021 | + | + | + | + | + | + | + | + | + |
| Oh et al., (61) 2019 | + | - | + | + | + | - | + | - | - |
| Okada et al., (62) 2012 | + | + | + | + | + | + | + | + | + |
| Pareek et al., (63) 2020 | + | + | + | + | + | + | + | + | + |
| Paul et al., (64) 2023 | - | + | + | + | - | + | + | - | - |
| Pham et al., (65) 2021 | + | + | + | + | + | + | + | + | + |
| Sauneuf et al., (66) 2020 | - | + | + | + | + | + | + | - | + |
| Schriefl et al., (67) 2022 | + | + | + | + | + | + | + | + | + |
| Shibahashi et al., (68) 2020 | + | + | + | + | + | + | + | + | + |
| Shih et al., (69) 2019 | - | + | + | + | + | + | + | - | + |
| Song et al., (70) 2021 | + | + | + | + | + | + | + | + | + |
| Tsuchida et al., (71) 2021 | + | + | + | + | + | + | + | + | + |
| Vedamurthy et al., (72) 2021 | + | + | + | - | + | + | + | - | + |
| Wang et al., (73) 2019 | + | + | + | + | + | + | + | + | + |
| Weiss et al., (74) 2015 | + | + | + | + | + | + | + | + | + |
| Wu et al., (75) 2022 | + | + | + | + | + | + | + | + | + |
| Yoon et al., (76) 2018 | + | + | + | + | + | + | + | + | + |
