# Supplementary material for: Prediction performance of scoring systems after out-of-hospital cardiac arrest: A systematic review and meta-analysis
Source: PLoS One. 2024 Feb 1;19(2):e0293704. doi: 10.1371/journal.pone.0293704 (PMC10833585; doi:10.1371/journal.pone.0293704)
Supplement: S5 Table — (DOCX) [file pone.0293704.s009.docx]

**S5 Table. Statistical, practical and overall performance of neurological prediction scores (at hospital discharge).**

| **Ranking** | **Diagnostic performance (pooled AUC)** | **Fastest available predictors at admission** | **Easy to calculate** | **Overall** |
| --- | --- | --- | --- | --- |
| **1.** | CAHP score (0.876) | C-GRApH score, CAHP score | C-GRApH score | CAHP score |
| **2.** | OHCA score (0.840) | OHCA score | CAHP score | C-GRApH score |
| **3.** | C-GRApH score (0.764) | - | OHCA score | OHCA score |

*AUC: area under curve; CAHP: Cardiac Arrest Hospital Prognosis; OHCA: out-of-hospital cardiac arrest.*
