# Supplementary material for: Prediction performance of scoring systems after out-of-hospital cardiac arrest: A systematic review and meta-analysis
Source: PLoS One. 2024 Feb 1;19(2):e0293704. doi: 10.1371/journal.pone.0293704 (PMC10833585; doi:10.1371/journal.pone.0293704)
Supplement: S1 File — (DOCX) [file pone.0293704.s010.docx]

**S1 File. Searching strategy.**

**Pubmed**

Search: ('out-of-hospital cardiac arrest' OR 'OHCA') AND ('MIRACLE' OR 'OHCA' OR 'CAHP' OR 'C-GRAPH' OR 'SOFA' OR 'APACHE' OR 'SAPS' OR 'SWAP' OR 'TTM' OR score OR prediction OR prognostic*)

("out of hospital cardiac arrest"[MeSH Terms] OR ("out of hospital"[All Fields] AND "cardiac"[All Fields] AND "arrest"[All Fields]) OR "out of hospital cardiac arrest"[All Fields] OR ("out"[All Fields] AND "hospital"[All Fields] AND "cardiac"[All Fields] AND "arrest"[All Fields]) OR "out of hospital cardiac arrest"[All Fields] OR "ohca"[All Fields]) AND ("miracle"[All Fields] OR "miracles"[All Fields] OR "ohca"[All Fields] OR "cahp"[All Fields] OR "c graph"[All Fields] OR "sofa"[All Fields] OR ("apach"[All Fields] OR "apache"[MeSH Terms] OR "apache"[All Fields] OR "apaches"[All Fields]) OR "saps"[All Fields] OR "swap"[All Fields] OR "ttm"[All Fields] OR ("score"[All Fields] OR "score s"[All Fields] OR "scored"[All Fields] OR "scores"[All Fields] OR "scoring"[All Fields] OR "scorings"[All Fields]) OR ("predict"[All Fields] OR "predictabilities"[All Fields] OR "predictability"[All Fields] OR "predictable"[All Fields] OR "predictably"[All Fields] OR "predicted"[All Fields] OR "predicting"[All Fields] OR "prediction"[All Fields] OR "predictions"[All Fields] OR "predictive"[All Fields] OR "predictively"[All Fields] OR "predictiveness"[All Fields] OR "predictives"[All Fields] OR "predictivities"[All Fields] OR "predictivity"[All Fields] OR "predicts"[All Fields]) OR "prognostic*"[All Fields])

Translations

'out-of-hospital cardiac arrest': "out-of-hospital cardiac arrest"[MeSH Terms] OR ("out-of-hospital"[All Fields] AND "cardiac"[All Fields] AND "arrest"[All Fields]) OR "out-of-hospital cardiac arrest"[All Fields] OR ("out"[All Fields] AND "hospital"[All Fields] AND "cardiac"[All Fields] AND "arrest"[All Fields]) OR "out of hospital cardiac arrest"[All Fields] 'MIRACLE': "miracle"[All Fields] OR "miracles"[All Fields] 'APACHE': "apach"[All Fields] OR "apache"[MeSH Terms] OR "apache"[All Fields] OR "apaches"[All Fields] score: "score"[All Fields] OR "score's"[All Fields] OR "scored"[All Fields] OR "scores"[All Fields] OR "scoring"[All Fields] OR "scorings"[All Fields] prediction: "predict"[All Fields] OR "predictabilities"[All Fields] OR "predictability"[All Fields] OR "predictable"[All Fields] OR "predictably"[All Fields] OR "predicted"[All Fields] OR "predicting"[All Fields] OR "prediction"[All Fields] OR "predictions"[All Fields] OR "predictive"[All Fields] OR "predictively"[All Fields] OR "predictiveness"[All Fields] OR "predictives"[All Fields] OR "predictivities"[All Fields] OR "predictivity"[All Fields] OR "predicts"[All Fields]

**Embase**

('out-of-hospital cardiac arrest'/exp OR 'out-of-hospital cardiac arrest' OR 'ohca'/exp OR 'ohca') AND ('miracle'/exp OR 'miracle' OR 'ohca'/exp OR 'ohca' OR 'cahp' OR 'c-graph' OR 'sofa'/exp OR 'sofa' OR 'apache'/exp OR 'apache' OR 'saps'/exp OR 'saps' OR 'swap' OR 'ttm' OR 'score'/exp OR score OR 'prediction'/exp OR prediction OR prognostic*)

**Scopus**

TITLE-ABS-KEY (('out-of-hospital AND cardiac AND arrest' OR 'ohca' ) AND ('miracle' OR 'ohca' OR 'cahp' OR 'c-graph' OR 'sofa' OR 'apache' OR 'saps' OR 'swap' OR 'ttm' OR score OR prediction OR prognostic*))

**Cochrane**

('out-of-hospital cardiac arrest' OR 'OHCA') AND ('MIRACLE' OR 'OHCA' OR 'CAHP' OR 'C-GRAPH' OR 'SOFA' OR 'APACHE' OR 'SAPS' OR 'SWAP' OR 'TTM' OR score OR prediction OR prognostic*)
